# Peer-reviewed by human experts: AI failed in key steps to generate a scoping review on the neural mechanisms of cross-education

**DOI:** 10.1007/s00421-025-06100-w

**Published:** 2025-12-24

**Authors:** M. Morrone, T. Hortobágyi, D. Kidgell, J. P. Farthing, F. Deriu, A. Manca

**Affiliations:** 1 Department of Biomedical Sciences, University of Sassari, Sassari, Italy; 2 Department of Kinesiology, Hungarian University of Sports Science, Budapest, Hungary; 3 Department of Human Movement Sciences, University Medical Center Groningen, University of Groningen, Groningen, The Netherlands; 4 Monash Exercise Neuroplasticity Research Unit, School of Primary and Allied Care, Monash University, Frankston, Australia; 5 College of Kinesiology, University of Saskatchewan, Saskatoon, Saskatchewan Canada; 6 Endocrinology Unit, AOUSS Sassari, Sassari, Italy

**Keywords:** Generative AI, Plagiarims, Scholarly Publishing, Peer review, Evidence synthesis, Neurophysiology

## Abstract

The integration of Large Language Models (LLMs) into scientific writing presents significant opportunities for scholars but also risks, including misinformation and plagiarism. A new body of literature is shaping to verify the capability of LLMs to execute the complex tasks that are inherent to academic publishing. In this context this study was driven by the need to critically assess LLM’s out-of-the-box performance in generating evidence synthesis reviews. To this end, the signature topic of the authors’ group, cross-education of voluntary force, was chosen as a model. We prompted a popular LLM (Gemini 2.5 Pro, Deep Research enabled) to generate a scoping review on the neural mechanisms underpinning cross-education. The resulting unedited manuscript was submitted for formal peer-review to four leading subject-matter experts. Their qualitative feedback on manuscript’s structure, content, and integrity was collated and analyzed. Peer-reviewers identified critical failures at fundamental stages of the review process. The LLM failed to: (1) identify specific research questions; (2) adhere to established methodological frameworks; (3) implement trustworthy search strategies; (4) objectively synthesize data. Importantly, the Results section was deemed interpretative rather than descriptive. Referencing was agreed as the worst issue being inaccurate, biased toward open-access sources (84%), and containing instances of plagiarism. The LLM also failed to hierarchize evidence, presenting minor or underexplored findings as established evidence. The LLM generated a non-systematic, poorly structured, and unreliable narrative review. These findings suggest that the selected LLM is incapable of autonomously performing scientific synthesis and requires massive human supervision to correct the observed issues.

**Supplementary Information:**

The online version contains supplementary material available at 10.1007/s00421-025-06100-w.

## Background

One of the most impactful innovations integrated in generative artificial intelligence (GenAI) is natural language processing (Leslie and Rossi 2023). As trained on a gigantic amount of text, GenAI enables communication between computers and humans. The software then understands and generates human language (Kacena et al. 2024). When a transformer type of neural network architecture sequentially processes a massive amount of textual data, a Large Language Model (LLM) can be generated. LLMs can communicate with humans reacting to prompts by forecasting suitable text responses based on training data as input (Hutson 2022).

LLMs are now being used in medical research and scientific writing, primarily because LLMs can perform tasks instantly that would otherwise require significantly more human effort and time (Huang and Tan 2023). LLMs’s rapid adoption by scholars in publishing has prompted considerable debate. especially when an LLM is used to write a full paper or when it is listed among the authors (Lee 2023).

Using GenAI to create educational or medical content raises risks about text integrity, validity, comprehensiveness, language quality and referencing. Without human verification, LLMs may include false but convincing information, “hallucinations” (Camarata et al. 2025), Such information gives rise to concerns regarding plagiarism, copyright infringements, ethical implications, and, most importantly, misinformation. Despite the continuous refinement of these models to improve output quality, and the expected enhanced utility due to a broader adoption by academics, the present challenges remain. In response to the growing influx of unsupervised LLM-generated manuscripts flagged by journal editors and peer reviewers, many scientific outlets have updated submission guidelines that now require authors to disclose the use of GenAI. The instructions also encourage authors to include the prompts and outputs as supplementary files. A brand-new body of knowledge is emerging around this topic in the form of analyses, reviews and editorials addressing? with the consequences of GenAI on research integrity. Examining LLMs’ capabilities in assisting scholars in the complex process of generating publishable scientific seems a worthy endeavour, especially if the findings generated will prove of practical utility to foster regulatory practices about using this technology in research.

To this end, we prompted an LLM-generated review on a topic of our foremost interest, the neural mechanisms underpinning cross-education. We then asked leading experts in this field to critically peer-review the LLM-generated review. Cross-education, the increased motor output of the opposite, untrained limb following a period of unilateral exercise training (Manca et al. 2021), is a widely studied phenomenon and a common neurophysiological model to investigate interhemispheric connectivity. Because the scientists here involved are recognized experts of the neurophysiological mechanisms underlying inter-limb transfer of force or a motor skill, the choice of the specific topic for a scoping review was suitable to: (1) determine if LLM produced a factually correct reflection of the state-of-the-art on the topic; (2) ascertain if LLM succeeded in presenting the results in a hierarchically organized manner according to the strength of the evidence; (3) assess if LLM cited the pertinent literature comprehensively and accurately, and (4) verify if evidence-based, worth-considering directions for future research were streamlined, as expected from a scoping review (Arksey and O’Malley 2005).

## The prompt

Box 1 reports the LLM-script title and the prompt that was used to generate the review in Gemini 2.5.5 Pro (03/25 model, Deep Research enabled, Google, 2025; https://gemini.google.com/ accessed April, 11th 2025). We utilized the standard web interface settings (Temperature = 1.0; dynamic Top-P and Top-K parameters not disclosed by the platform). To guide the LLM and optimize its operation, we devised the prompt to mimic a search performed by an experienced researcher. To assess the model’s autonomous capabilities, we employed a single-shot prompting strategy: the prompt was not further refined or adjusted based on the initial result from the LLM. To control for generation variability, we executed the prompt three times under identical conditions. One iteration produced an incomplete review; the remaining two were structurally and semantically consistent. Consequently, the first of the complete generations was selected for expert evaluation.

Box 1 **Prompt built to generate the review in Gemini 2.5 Pro 03/25 model, Deep Research enabled, Google, 2025;** https://gemini.google.com/**accessed April, 11th 2025**

**Task:**

- Generate a comprehensive review that maps the current evidence, identifies major gaps and limitations, and outlines potential directions for future research on the neural adaptations that mediate cross-education of strength.
- Focus on accuracy, take as much time as you need to.

**Title:**

- Neural mechanisms mediating cross-education of voluntary force: a scoping review mapping main gaps, limitations and future directions for research

**Background and Context:**

The phenomenon of cross-education refers to the observation that unilateral motor training can lead to performance improvements in the contralateral, untrained limb. This phenomenon is also known in the literature by several synonyms, including:

- Cross education
- Interlimb transfer
- Cross-training
- Contralateral training effect
- Contralateral strength training effect
- Contralateral effect

The objective is to thoroughly review and synthesize the existing scientific evidence on the neural mechanisms of cross-education, while also critically evaluating methodological limitations and identifying unexplored research directions.

**Requirements:**

Literature Selection:

- Use only peer-reviewed scientific articles sourced from PubMed, Google Scholar, Scopus, and Web of Science.
- Ensure that the review captures the most up-to-date and relevant studies, using the synonyms provided to broaden

the search scope.

**Structure and Content:**

- **Abstract:** A succinct summary of the review’s scope, key findings, and implications.
- **Introduction:** Overview of the phenomenon of cross-education, its significance in physiology, and the rationale for a scoping review.
- **Methodology:** Detailed description of the search strategy, including databases used, search terms (including all synonyms), inclusion/exclusion criteria, and any quality assessment methods.
- **Results:** Comprehensive mapping of the current evidence. Summarize key findings on the neural mechanisms involved in cross-education.
- **Discussion:** Critical analysis of the identified gaps and limitations in current literature. Discuss discrepancies, methodological constraints, and areas where evidence is lacking.
- **Future Directions:** Propose specific research directions and methodological improvements that could address the identified gaps.
- **Conclusion:** Summarize the major insights and the implications of the findings for future research.

**Tone and Style:**

- Use an academic and analytical tone. Provide a balanced discussion supported by recent and high-quality scientific evidence. Ensure clarity, precision, and appropriate citation of all sources.

**Additional Instructions:**

- Be critical and integrative in your synthesis of literature. Adhere strictly to the scientific rigor expected in a peer-reviewed review.

## Peer-reviewing the LLM-generated article

Supplementary Material 1 presents the full-text article generated by the LLM. We distinctly label this document in the header and footer of each page: “AI-WRITTEN ARTICLE”. On the right side of each page, we display the comments made by the experts who peer-reviewed the LLM-review. We asked the experts to review the articles blind to the other experts’ comments. All their annotations, remarks and feedback are reported in full version in the AI-written article. Starting from this document and after consultation among the article curators, major issues from each expert were identified, extracted and attributed to experts by consensus. Table 1 summarizes the Reviewers’ main feedback as a result of this process. The sections below summarize the experts’ assessments of the LLM-generated article.

**Table 1:** Summary of Reviewers’ feedback on the AI-generated scoping review on the neural mechanisms underpinning cross-education of voluntary force

Section of AI-Review	Reviewer 1	Reviewer 2	Reviewer 3	Reviewer 4
Abstract	Content is correct but has grammatical and phrasing errors.	Too many abbreviations.	Doesn’t resemble a scoping review abstract (lacks study counts, evidence quality).	Readable but too many unexplained abbreviations
		Reports underexplored findings as consolidated knowledge.	Inaccurate references to neurophysiological protocols.	Lacks hierarchy; minor findings are given the same weight as major ones.
		Insufficiently detailed to guide future research.		
Introduction	Comprehensive, but citations are problematic and don’t always support the statements. Warrants careful checking of all references.	Suboptimal or inappropriate referencing (relies on reviews, not primary sources).	Fails to detail training methods.	References are not original sources.
			Uses single references for long statements.	
		Lacks specificity (e.g., doesn’t specify upper/lower limbs).	Misses key papers from highly reputable, subscription-based journals.	Cites only open-access journals, missing seminal studies from subscription-based ones.
Rationale & Objectives	Aims are disconnected from the review’s practical application focus.	Strongly criticizes this section.	No negative or positive remarks on this subsection	Critical failure: Did not articulate specific research questions, a defining stage of scoping reviews.
		Aims are overly broad, vague, and disconnected from the background.		
		Fails to define key terms like "controversies."		
Methods	References cited to support the methodology are unsuitable.	Incorrect or inappropriately applied references.	Falsely claims adherence to PRISMA-ScR guidelines.	No PRISMA flow chart.
		Doesn’t adhere to seminal scoping review frameworks.	Contradicts its own criteria by relying on secondary sources (reviews) while claiming to exclude them.	Lacks transparency; makes false claims about database searches and reviewer calibration.
		Arbitrary literature cut-off date, missing recent key studies.		Suggests a major flaw is the AI’s inability to access pay-walled academic databases.
Results	Fails to report key data (e.g., number of studies).	Lacks detail, structure, and a PRISMA flow diagram.	Notes the AI provides a “template” for a review ("insert diagram here").	Includes irrelevant studies (motor skills instead of force).
	Raises concerns of plagiarism/copyright infringement.	Mixes results with discussion and interpretation, which is inappropriate.	Referencing is mostly inadequate or wrong.	Total absence of quantitative data (e.g., number of studies per technique).
	Praised some summaries (e.g., on TMS) but notes the AI used a preliminary, uncorrected version of a cited paper.	Contains blatant plagiarism (e.g., "insert flow diagram here").	Attributes findings to incorrect sources.	Mixes results with discussion, making it hard to follow.
		No summary tables to structure the findings.	Elaborates on topics using studies unrelated to cross-education.	
Discussion	Highly repetitive and redundant.	Repetitive and disconnected from the results; introduces new topics not previously covered.	Repetitive yet did a decent job identifying some key research gaps.	Repetitive; issues stem from poor objectives and results.
	Discusses methodological limitations but fails to connect them to the review’s focus on neural adaptations.	Tone is interpretive, not descriptive as required for a scoping review.	Noted the use of generic, non-substantive statements that could apply to any research field.	Disconnected from the evidence presented.
		Recommendations are overly ambitious and impractical.		Lack of hierarchization; minor/inconsistent findings are given disproportionate coverage.
		Structure lacks coherence.		
References	Countless inaccuracies and errors in the background and, overall, in the whole manuscript.	All references need to be reviewed for accuracy and position within the text, demanding hours and hours of *human* time.	Numerous issues with accuracy, duplication of the same source that was retrieved from different platforms (e.g., pre-print server, journal source, repositories). Reliance on review articles	Critical failure: inaccuracies permeating the whole bibliography, including all the call-outs within the text, threatening the review’s trustworthiness and reliability. The LLM is below-the-bar in managing scientific bibliography.

### ‘Abstract’

The abstract readability was found to be affected by compound, repetitive or awkward phrasing, and several unnecessary abbreviations. While the information summarized was deemed “broadly accurate”, the abstract was also described as “insufficiently detailed to support specific recommendations for future research directions.” Moreover, it “did not look like the summary of a scoping review, which should include the number of previous pertinent studies and the width and quality of the evidence.” Additionally, some of the findings reported as consolidated knowledge were, instead, inconsistent in the literature, or markedly underexplored, with minor adaptations portrayed likewise established mechanistic pillars of cross-education (*e.g*., increased cortical excitability and reduced intracortical inhibition of the “untrained” hemisphere).

### ‘Introduction’

While being comprehensive, as expected from “a reasonably well-trained LLM,” experts raised concerns about the review’s coherence between statements and cited references (*e.g.*, methodological requirements for planning cross-education studies based on a very small case series from Morrone et al. 2025), warranting careful checking of each citation. The seminal and most influential experimental studies on cross-education mechanisms published in highly reputable subscription-based journals, were omitted, since the references cited tended to be published in open-access only journals.

Another problematic aspect of the Introduction related to a general lack of specificity permeating the whole background (*e.g*., presenting percent magnitude of inter-limb transfer without specifying whether it referred to upper or lower limbs), preventing the reader from accurately interpreting and contextualizing the findings.

### ‘Rationale and objectives’

Rationale and objectives were found disconnected from the background because knowledge gaps, heterogeneity and inconsistencies among studies were not presented. The statement that the review “aims to systematically map the extent, range, and nature of research activity, identify key concepts, clarify working definitions, and pinpoint gaps in the existing literature” appeared as overly broad and generic, making the scope too vague to offer meaningful insights. Additionally, while ‘knowledge gaps’ and ‘inconsistencies’ were generally well understood, the term ‘controversies’ seemed ambiguous in this context as it did not specify whether ‘controversies” referred to conflicting theoretical models, divergent findings across studies, debates around neural mechanisms (*e.g.*, cortical *vs.* subcortical contributions), or disagreements regarding methodological standards, such as differences in training protocols, populations, outcome measures, or neural assessment techniques. Another issue was that the most defining stage of scoping reviews, *i.e.*, identification of the research question/s, was not performed despite the LLM-review declared adherence to state-of-the-art methodology for performing scoping reviews (Peters et al. 2015).

### ‘Methodology’

Most references supporting the review methodology were not methodological papers and specific statements were not supported by suitable evidence. For instance, reference #6 (a scoping review conducting by Voskuil et al. 2023) was cited to clarify the methodology behind scoping reviews even though it is not a source for scoping review methodology. Overall, the review framework did not adhere to seminal methodology (Arksey and O’Malley 2005; Levac et al. 2010; Peters et al. 2015) to complement the PRISMA-Scoping reviews (ScR) checklist (Tricco et al. 2018) and did not elaborate on how the selected review framework was operationalized to address the research question concerning neural mechanisms of cross-education. Relatedly, while the LLM-review claimed adherence to PRISMA ScR, many required aspects of the reporting of results and description of the search process were missing, indicating that adherence to PRISMA was rather formal. One example was the lack of a flow chart portraying screening and eligibility processes, as typically done when synthesizing the literature.

While generally broad in scope, the literature search strategy was indicated to be limited by an arbitrary cut-off date set at September 2024 (seven months before the LLM-review was generated, probably due to the cut-off point of the machine learning/deep learning training data), excluding the newest advancements in the neural determinants of cross-education, such as the two mechanistic works by Lecce et al. (2025a, b), and therefore threatening the currency and relevance of the review. Puzzlingly, and probably related to the Deep Research function, these studies were then mentioned in the Results section, in a table summarizing the evidence for proposed neural mechanisms mediating cross-education of force.

A concerning lack of trustworthiness of the declared search methodology was highlighted, as “*...supplementary databases sometimes cited in related reviews were also ****considered or implicitly covered**** through the primary databases.*” However, such databases were not accessed, possibly because these are pay-walled and/or subscription-based platforms that are typically accessible to scholar institutions. This may imply that running LLM inside or outside of an academic institution would make a difference in the final LLM-script, depending on the portfolio of publishers and journals subscribed by the scholar’s institution. This also implies that the LLM-generated article may be different if launched at work or home, although simulations are needed to test this hypothesis. Another threat to the credibility of LLM-generated articles relates to evident falsities, for instance: “*The screening process was conducted systematically, potentially involving ****calibration between reviewers**** on a subset of articles to ensure consistency statements*” or “...*Reference lists of included articles and relevant review papers were also ****manually**** screened to identify any additional eligible studies missed by the electronic search*.” It is evident that LLMs do not involve or liaise with any reviewers, and these sentences, which are typically part of reviews and meta-analyses, were basically extracted from another review article on cross-education.

### ‘Results’

Overall, the Results section was described by the reviewers as closer to a discussion than to a neutral presentation of the review findings.

The results section lacked descriptive objectivity, structural coherence, detail, and visual support expected in a scoping review. While the general summary provided a broad overview of the studies included, it omitted key quantitative and descriptive information, such as study design, sample sizes, training protocols, and outcome measures. The exact number of studies identified at each stage of screening was missing, and no PRISMA-ScR flow diagram was included to transparently illustrate the selection process. When dealing with the neurophysiological techniques and methods used to study cross-education, quantitative estimates were absent and the number of studies that have employed them was not reported. The sentence typically found throughout the results was that “*Specifically, studies have reported significant adaptations in*...” - making the LLM-review vague and suboptimal in synthesizing literature and preventing the readers’ ability to get a feel of how wide or limited the evidence is in support of or against a certain mechanistic view.

Concerns of copyright infringement and plagiarism were raised, as the LLM wording resembled almost literally that of some of the text published by specific authors, in many instances from papers published by the same reviewers of the present work, without even referencing the articles. To this regard, the LLM-text included some hyperlinks pasted from other reviews and even indications such as “...*a PRISMA-ScR flow diagram illustrating this process ****could be inserted here***”, as an evident form of plagiarism. Moreover, this latter example reveals a double issue: while copying the phrase is a clear instance of plagiarism, the LLM did not attempt to create or mimic a diagram. Instead, it provided a structural placeholder, effectively acting as a template to guide the manuscript’s preparation.

A positive aspect that was agreed upon by the reviewers was the LLM capability to synthesize the complexity inherent to the interhemispheric dynamics underpinning cross-education, and the debate over the utility of transcranial magnetic stimulation measures made at rest to probe neural adaptations occurring during contraction. But, in doing so, it reported the contents of a preliminary uncorrected version of the work by Manca et al. (2018), even though the final version was available under the open access policy, rising further concerns about the references’ reliability and currency.

Issues in the quality of referencing were underlined also in this section as, for instance, the findings about interhemispheric inhibition were attributed to a secondary source (Altheyab et al. 2024) rather than the seminal experimental work (Hortobágyi et al. 2011) or other foundational papers published in subscription-based journals that the LLM could not access. Another notable aspect is that LLM elaborated on outcomes (*e.g.*, V-wave) mostly based on studies that did not study cross-education (Gomes et al. 2025), while missing a directly appropriate reference (*e.g.,* Colomer-Poveda et al. 2017).

### ‘Discussion’

The Discussion was utterly repetitive, since out-of-place extensive discussion, interpretation and elaboration were already present in the Results section. Another flaw of the LLM-review was reverberation of classical methodological limitations of cross-education studies that, however, in no way were related to the focus of the scoping review, *i.e*., neural adaptations.

Overall, the Discussion was disconnected from the Results and the mapped evidence. Rather than presenting a descriptive synthesis of the mapped evidence, LLM often adopted an interpretive tone, made causal inferences and proposed mechanistic models that were not directly supported by the included studies, often going beyond the scope of the reported data, not clearly qualifying them as hypothetical or speculative. Several topics, such as cerebellum and basal ganglia roles, or asymmetry in transfer effects, were discussed at length without adequate coverage in the results. This raises concerns about whether these areas were included in the original data extraction or were introduced *post hoc*, possibly because of extracting sentences from the discussion of cross-education papers/reviews. While methodological challenges, supraspinal and spinal mechanisms were addressed, they were not consistently aligned with the guiding questions of the scoping review. A clearer organizational framework reflecting the aims and pre-specified domains of interest would have improved the narrative and ensured a meaningful contribution of each section to answering the review’s core questions.

Another relevant issue was the lack of hierarchization of the review findings. Minor or under-replicated results were given as much or even more coverage than established aspects, as were the mixed findings about increased V-wave in the untrained muscles (Fimland et al. 2009; Colomer-Poveda et al. 2017; Bouguetoch et al. 2021), a relatively underexplored area studied in few cross-education studies. Relatedly, assertions that V-wave increases reflect greater descending drive were repeated without discussing opposing evidence or limitations inherent in V-wave interpretation. Similarly, the effects of eccentric training were highlighted as consistent, yet no meaningful discussion was provided on factors that may modulate them. In the same way, although the “Cross-Activation” and “Bilateral Access” models were widely cited, the section comparing them was oversimplified and under-referenced, and their relative empirical support was not adequately discussed. Anecdotal examples such as callosal agenesis were introduced without being part of the mapped evidence, weakening the argument and blurring the line between evidence synthesis and narrative review. A more critical appraisal of these nuances would have enhanced the credibility of the discussion. Finally, recommendations for future research were deemed comprehensive yet somewhat commonplace as LLM tended to recurrently produce generic statements that could be said about almost any area of research, such as: “*Adopting common data elements and standardized reporting checklists could significantly enhance the quality and interpretability of future research*.” Conversely, other recommendations seemed overly ambitious in places. Suggestions such as employing microneurography or advanced dynamic causal modelling may not be practical given the current state of the field. While these methods are valuable, their feasibility and relevance should be weighed more carefully, and recommendations should be grounded in the actual gaps identified in the reviewed literature.

### ‘References’

Of the sixty-two citations produced by LLM, 84% (52/62) were from open-access sources: journal websites (*n* = 26), PubMed Central (*n* = 14), ResearchGate (*n* = 6), university repositories (*n* = 5), and medRxiv (*n* = 1). One item from a university repository and the medRxiv item were preprints that differed meaningfully from the subsequently published open-access articles. Two citations referred only to Science.gov “topic” pages, preventing us from determining which underlying articles were intended or how they were selected. Among the eight non-open-access items, five linked to author profiles on ResearchGate; one pointed only to the abstract, and, for that same article, LLM also cited an earlier medRxiv version. LLM also duplicated several works across locations (*e.g*., journal site, PubMed Central) and treated each location as a distinct reference.

LLM depended on secondary syntheses, and many cited versions often corresponded to uncorrected author manuscripts or preprints with tracked changes; further problems included duplicated references and formatting inconsistencies that hampered retrieval of bibliographic details.

Overall, Reviewers concluded that LLM’s pervasively inaccurate referencing (such as mis-citations, under-citations, and several sentences left unreferenced) undermined the trustworthiness of the review and demonstrated that LLM is, at present, below the bar for reliable management of scientific references.

## Collating experts’ feedback into a unified appraisal

Taken together, the comments of leading scientists in the field reveal generally mixed feelings pointing toward an apparently *good job* in reviewing the literature at a first look, but several substantial weaknesses when the LLM-review is scrutinized more closely.

### Need for a credible Reference Manager

All reviewers found *References* as the most problematic section, citing significant inaccuracies in how articles were cited within the text and bibliography. These errors necessitated extensive human supervision and make the correction process a substantial undertaking. Inappropriate citation threatens a core pillar of scholarly publishing: contextualising research, demonstrating its breadth and depth, and properly acknowledging other people’s work.

This referencing failure must be distinguished as largely an ecosystem constraint rather than solely a model capability issue. Indeed, the problem cannot be disconnected from the open-access movement. Open-access publishing models are well-known and praised for making scientific knowledge freely available to users, increasing the diversity of the communities that benefit from it (Huang et al. 2024), which is now a priority for stakeholders and community. However, in the era of GenAI it also comes with a price. Indeed, the tested model accessed mostly those free-to-read open-access articles, almost totally neglecting papers that had been published in subscription-based journals before and after the global explosion of open-access. Accordingly, all the Reviewers complained that early foundational works published before and after the open-access advent, escaped the review, threatening its validity and trustworthiness.

The strategy employed by the model to include paywalled articles poses further problems, as some of them were accessed through sites and platforms connecting researchers like ResearchGate, which may lack rights to host full-texts, leading to possible copyright infringement. This problem persists because researchers tend to prefer publishing in prestigious subscription-based journals over publishing with the open-access model. This problem seems the easiest to address technically as Gemini 2.5 Pro can process very large amounts of text in a single session, up to one million tokens. Therefore, context size is only a minor constraint and not the main reason for the referencing problems described above. Addressing this issue requires broader open-science initiatives and formal agreements between AI developers and publishers, not simply larger context windows. In the emerging “zero-click” era, in which users increasingly rely on the model’s response rather than visiting the source, the inaccessibility of subscription-based research threatens both the reliability of AI-generated reviews and the circulation of scientific knowledge.

### Need to assess the quality of evidence and hierarchize findings

Another of the Reviewers’ concerns mostly threatening the educational reach of the LLM-review is the lack of hierarchy in the relevance of the reported findings: minor findings are given the same weight of established knowledge, which negates one of the pillars of scholarly education where evidence must be appraised, weighted and ranked. All Reviewers agreed on the need for human supervision and rewriting, although they also admitted that such a process would require immense effort for content verification, attribution, contextualization and interpretation. The question is: would this work require more time than just writing the paper ourselves?

This limitation in how evidence is appraised and ranked is not inherent to the technology itself as it may stem from how current user-facing systems are built. Technically, solutions like developing literacy in pipelines like the Retrieval-Augmented Generation that provide models with structured metadata (such as study design, sample size, and journal quality, citations, etc.), or design multi-agent workflows, would allow scholars to use current LLMs more responsibly and offset some of their weaknesses.

The greater responsibility lies with LLM providers, who should embed evidence-based mechanisms into model design, making them accessible not just to technically skilled researchers. Integrating established frameworks such as GRADE (Grading of Recommendations, Assessment, Development and Evaluations), would guide models to systematically evaluate risk of bias, consistency, and precision. Incorporating such methods during fine-tuning or developing them into dedicated scientific LLMs, could deliver systems capable of producing comprehensive and scientifically rigorous reviews. Progress requires, however, a joint action: researchers adopting these methods to make better use of existing tools, and providers embedding them at scale so that reliable, evidence-weighted synthesis becomes the standard rather than the exception.

### Need to go beyond reiteration

Especially in the *Conclusions* section, the review reiterated broad theoretical mechanisms rather than synthesizing the mapped evidence. Known concepts were restated without being linked to number, type, or quality of included studies. In this sense, LLM-review is *reproducing* (approaching plagiarism and without attribution) rather than *producing* a synthesis and the related roadmap. Overall, the LLM-review reverberates key messages conceptualized and demonstrated elsewhere, but what is really missing is that the process did not lead to identify benchmarks to understand the neural mechanisms of cross-education, which would advance the knowledge in the field. Would we understand the mechanism more accurately if we could predict response magnitude from data collected at the baseline? The LLM does not say anything about which benchmark to use to express the level of understanding of the mechanisms. Would it be a multiple regression or some other analysis in which 90% of variation in cross-education would be explained by, for instance, six or less neural predictors? The LLM managed to pull together the existing data but provided no new, creative insights into exploring what is the measure/benchmark/marker of understanding the neural mechanisms of the transfer both conceptually (neuroscientifically) and behaviorally. These are complex interpretative tasks that currently remain in the exclusive domain of *humans*.

## Final remarks on the LLM-review

A key limitation of this study is its intentionally narrow focus on cross-education mechanisms in healthy individuals, the authors’ main research area. While this provided a controlled framework to test the LLM’s ability to generate a scientific review, it also restricted the study’s scope and generalizability to pathological conditions. We also acknowledge the dual role of the authors as both investigators and subject-matter experts. While expertise in this domain was critical for identifying subtle factual errors and hallucinations that a generalist might miss, it introduces a potential interpretive bias. To mitigate this, we prioritized objective evaluation metrics such as the verification of reference existence, data accuracy, and hierarchy of evidence, over subjective stylistic assessments.

Moreover, our prompt was deliberately designed to resemble the search and writing approach of a senior researcher with domain expertise in physiology but without GenAI expertise, and we did not attempt to improve or alter the prompt based on what the results were. These choices reflect the scope of our project, which was to evaluate the Deep Research out-of-the-box capabilities, rather than best-case performance achievable through expert prompt engineering, multi-agent frameworks, or fine-tuning.

Looking ahead, it is reasonable to expect that outcomes could improve substantially if the same task were attempted by an expert user of LLMs and by editing or improving the prompt after evaluating the quality of the product. Combining domain knowledge with technical strategies might enable the model to generate more structured, evidence-weighted, and benchmark-oriented reviews. Future LLM-assisted reviews may therefore require the same dual expertise that underpins traditional systematic reviews, where subject-matter specialists work alongside methodological experts and statisticians to ensure rigor and reliability. Another avenue of development would be launching the prompt over different LLMs and then comparing capabilities.

## Conclusions

The main finding of this analysis is that this LLM-generated scoping review did not perform key steps of the framework that is commonly adopted for conducting a scoping review (Arksey and O’Malley 2005), *i.e*., identifying the research questions, selecting studies to be included in the review comprehensively, charting the data, collating, summarizing and reporting the results of such a process. Instead, it proved to be a narrative, non-systematic poorly structured review. This conclusion doesn’t mean that scholars should give up on the use of LLMs for scientific writing.

With these considerations in mind, the question is what can we all do to implement this technology to make it as fair, ethical and useful as possible? Because GenAI has now entered our lives to stay, no efforts should be spared to make this new technology better, especially in the educational realm.

In conclusion, even though LLMs seem still unable to replace subject matter experts, they promise to transform how reviews are prepared, “*...shifting the human role from exhaustive curator to creative synthesizer, empowered by intelligent, always-on review-copilots*" as neatly envisioned by Zhiling Zhang in his *Nature* commentary titled "*The future of reviews writing in the AI era*” (Zheng 2025).

## Supplementary Information

Below is the link to the electronic supplementary material.

Supplementary file1 (DOCX 6298 KB)

## Data Availability

No data have been analyzed for the present article as this document summarizes previous evidence.
