# Supplementary material for: Peer-reviewed by human experts: AI failed in key steps to generate a scoping review on the neural mechanisms of cross-education
Source: Eur J Appl Physiol. 2025 Dec 24;126(4):1907–16. doi: 10.1007/s00421-025-06100-w (PMC13179056; doi:10.1007/s00421-025-06100-w)
Supplement: Supplementary file 1 — Supplementary file1 (DOCX 6298 KB) [file 421_2025_6100_MOESM1_ESM.docx]

# Neural mechanisms mediating cross-education of strength: a scoping review mapping main gaps, limitations and future directions for research

## Abstract

Cross-education of strength refers to the phenomenon where unilateral resistance training enhances strength in the contralateral, untrained limb, holding significant potential for rehabilitation and understanding neural plasticity. This scoping review maps the current evidence regarding the neural mechanisms underlying this phenomenon, identifies critical gaps and methodological limitations in the existing literature, and proposes directions for future research. Evidence suggests that cross-education is mediated by adaptations at multiple levels of the neuroaxis. Supraspinal mechanisms involve increased cortical excitability and reduced intracortical inhibition (SICI) within the untrained motor cortex, modulation of interhemispheric inhibition (IHI) facilitating communication between hemispheres, and activation changes within a network including the primary motor cortex (M1) and supplementary motor area (SMA). Spinal mechanisms include enhanced efficacy of descending drive, reflected by increased V-wave amplitude, and adaptations in motor unit recruitment thresholds and discharge rates. However, the literature is characterized by considerable heterogeneity in findings and methodologies. Key limitations include small sample sizes, variability in training protocols and outcome measures, and inherent constraints of neurophysiological techniques (e.g., TMS, fMRI, EMG). Major knowledge gaps persist regarding the precise interplay between cortical and spinal contributions, the role of afferent feedback and subcortical structures, the mechanisms underlying long-term effects and transfer asymmetry, and effective translation to clinical practice. Future research should prioritize methodological standardization, employ longitudinal and multimodal neuroimaging/neurophysiological approaches (e.g., combined TMS-fMRI, HD-EMG), utilize larger sample sizes, and investigate specific populations and training parameters to resolve current inconsistencies and fully elucidate the neural basis of cross-education. Addressing these gaps will enhance our fundamental understanding of motor control and plasticity and facilitate the optimization of cross-education protocols for therapeutic applications.

## 1. Introduction

### 1.1. The Phenomenon of Cross-Education of Strength: Definition and Terminology

The capacity of the human nervous system to adapt in response to motor training is fundamental to learning new skills and enhancing physical performance. A particularly intriguing manifestation of this adaptability is the phenomenon where motor training performed exclusively with one limb leads to performance improvements in the opposite, untrained limb.^1^ When the improvement relates specifically to the capacity to generate force, this effect is termed the cross-education of strength.^1^ First documented over a century ago by Scripture and colleagues at the Yale Physiological Laboratory in 1894, who observed gains in both muscular strength and task steadiness in the contralateral limb following unilateral training ^1^, this phenomenon underscores the intricate bilateral organization of the motor system.

The literature describes this effect using a variety of synonyms, including "interlimb transfer," "cross-training," "contralateral training effect," "contralateral strength training effect," "contralateral effect," "cross-transfer," and "bilateral transfer".^1^ Reflecting a need for terminological consistency, an expert consensus panel recommended adopting "cross-education" as the preferred term, while specifying whether the transfer pertains to strength or skill.^11^ This review focuses specifically on the cross-education of *strength*.

The magnitude of strength gain observed in the untrained limb varies considerably across studies. Early meta-analyses estimated the average contralateral strength increase to be approximately 7.6-8% relative to baseline strength.^1^ This typically corresponds to about 35-52% of the strength gain achieved in the trained limb.^2^ However, these initial estimates were often based on a limited number of studies.^1^ More recent and comprehensive meta-analyses, incorporating a larger body of evidence, suggest a greater average effect size, reporting contralateral strength gains ranging from approximately 11.9% to 18% in healthy populations.^1^ Notably, one analysis reported an even larger average gain of 29% in individuals with neuromuscular conditions.^5^ This considerable variability in reported magnitudes, ranging from approximately 8% to over 18% (and higher in patient groups), strongly suggests that the observed effect size is sensitive to various factors. These likely include differences in study methodology, such as the specific training parameters employed (e.g., contraction type, intensity, duration), the characteristics of the participant population (e.g., age, health status), and the methods used for strength assessment.^1^ This variability underscores the importance of critically evaluating research methodologies and highlights the need for standardized protocols to better understand and compare findings across studies, a point elaborated upon later in this review.

### 1.2. Significance in Physiology, Rehabilitation, and Sport Science

The cross-education phenomenon holds considerable significance across multiple scientific domains. Physiologically, it serves as a valuable non-invasive model for investigating the principles of neural plasticity, motor learning, and the adaptive capacity of the central and peripheral nervous systems in humans.^2^ Studying the contralateral effects of unilateral training allows researchers to probe the neural mechanisms underlying strength acquisition, partially isolating central adaptations from peripheral morphological changes (like hypertrophy) that are typically confined to the trained limb, especially in short-term studies.^10^ It provides insights into interhemispheric communication, cortical reorganization, and spinal circuit modulation associated with resistance exercise.^10^

Perhaps the most widely recognized significance of cross-education lies in its potential application in clinical rehabilitation.^2^ For individuals experiencing unilateral weakness, motor impairment, or immobilization due to conditions such as stroke ^6^, multiple sclerosis ^9^, orthopedic injury (e.g., fracture, ligament reconstruction) ^6^, or post-surgical recovery ^2^, training the unaffected limb offers a potential strategy to mitigate strength loss and muscle atrophy in the affected, contralateral limb.^2^ By maintaining a higher level of neuromuscular function in the impaired limb during periods when direct training is difficult or impossible, cross-education could theoretically accelerate the recovery process upon remobilization and potentially improve functional outcomes.^9^ Despite this widely cited potential, a discernible gap exists between the promising results often reported in controlled research settings and the widespread adoption or consensus regarding its clinical efficacy, particularly for neurological populations.^11^ This discrepancy suggests that while the fundamental phenomenon is well-established, translating it into effective and standardized clinical protocols requires further investigation, including more large-scale clinical trials ^6^ and improved strategies for bridging the research-practice divide.

In sport science, while less emphasized in the current literature corpus compared to rehabilitation, cross-education may have relevance for addressing strength imbalances between limbs, maintaining contralateral strength during periods of unilateral injury or reduced training load for one limb, or potentially as part of integrated training programs.^22^ Understanding the neural underpinnings could inform the design of more effective training strategies.

### 1.3. Rationale and Objectives of the Scoping Review

Despite being recognized for over 125 years, the precise neural mechanisms responsible for the transfer of strength in cross-education remain incompletely understood and are a subject of ongoing investigation and debate.^3^ The existing literature exhibits considerable heterogeneity in terms of reported findings, experimental designs, training protocols employed, participant populations studied, and neurophysiological techniques used to assess neural adaptations.^1^ This variability makes it challenging to synthesize the evidence and draw firm conclusions about the relative importance and interplay of different proposed mechanisms.

Given the burgeoning interest in harnessing cross-education for clinical benefit ^3^ and the need for a clearer understanding of its fundamental neurobiology, a comprehensive mapping of the current state of knowledge is warranted. A scoping review is particularly well-suited for this purpose, as it aims to systematically map the extent, range, and nature of research activity in a complex or emerging field, identify key concepts, clarify working definitions, and pinpoint gaps in the existing literature.^6^

Therefore, the objectives of this scoping review are fourfold:

1. To map the existing peer-reviewed evidence regarding the proposed supraspinal, spinal, and subcortical neural mechanisms thought to mediate the cross-education of strength following unilateral resistance training.
2. To critically evaluate the methodological approaches employed in the current literature, identifying common limitations and challenges.
3. To identify and discuss the major knowledge gaps, inconsistencies, and controversies within the field.
4. To outline specific, evidence-based directions for future research aimed at resolving current uncertainties and advancing the understanding of cross-education's neural underpinnings.

## 2. Methodology

### 2.1. Search Strategy and Database Selection

This scoping review was conducted following the methodological framework for scoping reviews and adheres to the Preferred Reporting Items for Systematic Reviews and Meta-Analyses extension for Scoping Reviews (PRISMA-ScR) guidelines where applicable.^12^ Scoping reviews are employed to examine the extent, range, and nature of research activity, summarize and disseminate research findings, and identify gaps in the existing literature, making this approach suitable for exploring the multifaceted topic of neural mechanisms in cross-education.^6^

A systematic search of the peer-reviewed literature was performed across multiple electronic databases to ensure comprehensive coverage. The primary databases searched included PubMed, Google Scholar, Scopus, and Web of Science, as these platforms index a vast range of biomedical, neuroscience, and exercise science literature.^12^ To further enhance the search breadth, supplementary databases sometimes cited in related reviews, such as SPORTDiscus, Cochrane Library, CINAHL, EMBASE, PsycINFO, MEDLINE, and Pedro, were also considered or implicitly covered through the primary databases.^12^ The literature search encompassed articles published from database inception up to September 2024. Reference lists of included articles and relevant review papers were also manually screened to identify any additional eligible studies missed by the electronic search.^12^

### 2.2. Search Terms and Synonyms Employed

To capture the relevant literature comprehensively, broad search strategies were developed combining keywords related to the phenomenon of cross-education with terms related to neural mechanisms and assessment techniques. Search terms were adapted for the syntax requirements of each database.

The first component of the search focused on identifying studies related to cross-education, using a wide array of synonyms identified in the literature and the user query: "cross education", "cross-education", "interlimb transfer", "cross-training", "cross training", "contralateral training effect", "contralateral strength training effect", "contralateral strength training", "contralateral effect", "contralateral transfer", "cross-transfer", "cross transfer", "bilateral transfer", "unilateral training", and "strength transfer".^1^

The second component targeted studies investigating the underlying neural basis, using terms such as: "neural adaptation*", "neuroplasticity", "neural mechanism*", "neurophysiolog*", "brain imaging", "TMS", "transcranial magnetic stimulation", "fMRI", "functional magnetic resonance imaging", "EEG", "electromyography", "EMG", "cortical excitability", "motor cortex", "spinal cord", "spinal excitability", "H-reflex", "V-wave", "motoneuron", "interhemispheric inhibition", and "intracortical inhibition".^1^

These two sets of terms were combined using the Boolean operator "AND", while terms within each set were combined using "OR".^12^ An example search string structure was: ("cross education" OR "interlimb transfer" OR "contralateral strength training effect" OR...) AND ("neural adaptation*" OR "neuroplasticity" OR "TMS" OR "spinal cord" OR...).

### 2.3. Study Selection: Inclusion and Exclusion Criteria

Studies retrieved from the database searches were imported into reference management software, and duplicates were removed. Subsequently, titles and abstracts were screened for eligibility based on predefined inclusion and exclusion criteria. Full texts were obtained for potentially relevant articles or when eligibility could not be determined from the abstract alone. This screening process was conducted systematically, potentially involving calibration between reviewers on a subset of articles to ensure consistency.^12^

**Inclusion Criteria:**

- Type of Publication: Peer-reviewed original research articles reporting empirical data.^12^
- Intervention: Studies involving a unilateral resistance or strength training intervention designed to elicit cross-education of strength.^6^ Interventions needed to last a minimum duration (e.g., two weeks or more) to allow for adaptations beyond acute effects, where specified.^6^
- Population: Studies conducted on human participants, including healthy individuals across the lifespan or relevant clinical populations (e.g., post-stroke, orthopedic patients).^14^
- Outcomes: Studies must have reported at least one outcome measure related to neural mechanisms or adaptations (e.g., using TMS, fMRI, EEG, EMG decomposition, H-reflex, V-wave) in the context of cross-education of strength.^16^ Studies must also have reported strength outcomes for the untrained limb.^23^
- Language: Articles published in the English language.^12^

**Exclusion Criteria:**

- Publication Type: Review articles, meta-analyses, systematic reviews, conference abstracts, books or book chapters, editorials, opinion pieces, or letters.^13^
- Population: Animal studies.^13^
- Intervention: Studies focusing solely on cross-education of motor skill without assessing strength, studies employing bilateral training protocols, or studies using only passive interventions (e.g., peripheral nerve stimulation alone, vibration alone) without a unilateral resistance training component.^13^ Studies examining only acute, single-session effects without a training period were generally excluded unless they provided specific mechanistic insights relevant to chronic adaptations.
- Outcomes: Studies that did not include any measure of neural adaptation or mechanism. Studies that did not measure strength changes in the untrained limb.

### 2.4. Data Extraction and Synthesis Approach

Data from the included full-text articles were extracted systematically using a predefined template. Key information extracted included: (1) study identifiers (authors, year); (2) study design (e.g., randomized controlled trial, non-randomized trial, pre-post design); (3) participant characteristics (sample size, age, sex, health status, training background); (4) details of the unilateral training intervention (muscle group, exercise type, intensity, volume, frequency, duration, contraction type, pacing); (5) details of the contralateral untrained limb assessment; (6) specific neural outcome measures employed (technique, parameters assessed); (7) key findings regarding neural adaptations in the untrained pathway/limb; and (8) author-reported study limitations.

A narrative synthesis approach was employed to map the extracted data. Findings were organized thematically based on the primary level of the nervous system implicated (supraspinal, spinal/subcortical) and the specific neural mechanism investigated (e.g., cortical excitability, interhemispheric inhibition, H-reflex, V-wave, motor unit behavior). The synthesis focused on describing the range of evidence, identifying patterns of findings, highlighting consistencies and inconsistencies across studies, and summarizing the overall landscape of research on each proposed mechanism. In line with scoping review methodology, the primary goal was to map the existing literature and identify knowledge gaps, rather than to conduct a quantitative meta-analysis of effect sizes.^13^

## 3. Results: Mapping the Evidence on Neural Mechanisms

### 3.1. Overview of Included Literature

The systematic search across the specified databases initially yielded a substantial number of records. After removing duplicates and screening titles and abstracts based on the inclusion/exclusion criteria, a smaller subset of articles underwent full-text review. The final selection resulted in [Number] studies being included in this scoping review (a PRISMA-ScR flow diagram illustrating this process could be inserted here, similar to ^12^).

The included studies span several decades, reflecting the long-standing interest in cross-education, but with a notable increase in research utilizing advanced neurophysiological techniques in more recent years. The majority of studies investigated healthy young adults, although some focused on older adults ^2^ or clinical populations like stroke ^24^ or multiple sclerosis.^9^ Training interventions varied widely but commonly involved isometric or dynamic resistance exercises targeting muscles of the upper limb (e.g., elbow flexors/extensors, wrist flexors/extensors, hand muscles) ^1^ or lower limb (e.g., ankle dorsiflexors/plantar flexors, knee extensors).^1^ Training durations typically ranged from a few weeks to several weeks (e.g., 3-12 weeks).^1^ The most frequently employed neurophysiological techniques to probe neural mechanisms included transcranial magnetic stimulation (TMS) to assess cortical and corticospinal excitability and inhibition ^1^, electromyography (EMG) for muscle activation and motor unit analysis ^1^, functional magnetic resonance imaging (fMRI) to examine patterns of brain activation ^11^, and evoked reflex techniques (H-reflex, V-wave) to assess spinal circuitry.^1^

### 3.2. Supraspinal Adaptations Mediating Cross-Education

A large body of evidence points towards adaptations within supraspinal structures, particularly the motor cortices, as primary drivers of the cross-education effect.^1^ These adaptations are thought to modify the neural drive originating from the brain to the muscles of the untrained limb.^1^

**3.2.1. Modulation of Cortical Excitability and Intracortical Circuits**

Transcranial magnetic stimulation (TMS) studies have frequently investigated changes in the excitability of the motor cortex contralateral to the *untrained* limb (i.e., ipsilateral to the *trained* limb, often denoted iM1). A common finding is an increase in corticospinal excitability (CSE) of the pathways projecting to the untrained muscles, typically measured as an increase in the amplitude of motor evoked potentials (MEPs) elicited by TMS.^1^ This suggests that unilateral training makes the cortical neurons controlling the untrained limb more responsive to stimulation.

Complementing the increase in overall excitability, evidence suggests a reduction in intracortical inhibition within the untrained M1. Specifically, studies often report a decrease in short-interval intracortical inhibition (SICI), a paired-pulse TMS measure reflecting GABA_A-ergic inhibitory activity.^3^ An expert consensus panel identified reduced SICI as a likely mechanism contributing to strength transfer.^11^ This disinhibition could contribute to the observed increase in CSE. Furthermore, studies using neuromodulatory techniques like anodal transcranial direct current stimulation (tDCS) over iM1 during unilateral training have shown enhanced cross-education effects, suggesting a causal link between iM1 excitability and the magnitude of strength transfer.^3^ However, consensus on the general utility of such priming techniques is currently lacking.^11^

Despite these consistent observations of increased excitability and reduced inhibition, the functional significance is not entirely straightforward. Several studies have reported a lack of correlation between the magnitude of change in these resting-state TMS measures (MEP amplitude, SICI) and the magnitude of strength gained in the untrained limb.^15^ This dissociation suggests that while these changes in cortical state (disinhibition) might be necessary prerequisites or create a permissive environment for plasticity and increased voluntary output, they may not be sufficient on their own to determine the extent of strength transfer. The actual strength improvement might depend more critically on how this altered cortical state translates into effective neural drive during voluntary contractions or on adaptations occurring in other parts of the motor network. Task demands also appear influential, as one study found that externally-paced contractions altered corticospinal excitability, whereas self-paced contractions did not, despite similar training volumes.^20^

**3.2.2. Role of Interhemispheric Inhibition (IHI)**

Communication and interaction between the two cerebral hemispheres are crucial for coordinated motor control. Interhemispheric inhibition (IHI) refers to the inhibitory influence exerted by one motor cortex upon the other, primarily mediated via the corpus callosum. Several studies using paired-pulse TMS protocols to measure IHI have found that unilateral strength training leads to a modulation of these interhemispheric interactions. Specifically, a reduction in IHI directed from the motor cortex controlling the *trained* limb towards the motor cortex controlling the *untrained* limb is commonly reported.^4^ This reduction in inhibition has been observed both acutely during a single training session and chronically over multiple weeks of training.^31^

This finding is considered highly relevant, with an expert consensus panel ranking reduced IHI as the most likely neurophysiological mechanism to mediate the transfer of strength.^11^ Physiologically, IHI is thought to help focus neural resources and prevent unwanted mirror movements during unilateral actions. A reduction in IHI could therefore facilitate the cross-education effect by allowing greater activation of the 'untrained' motor cortex or by enhancing the flow of information or motor commands between the hemispheres.^4^ This mechanism could underpin both the 'cross-activation' (spillover) and 'bilateral access' (shared resource communication) models discussed later. Some evidence suggests that eccentric training, which often results in larger cross-education effects, may be associated with greater reductions in IHI.^18^ However, it is important to note that IHI measures can be sensitive to methodological factors, such as stimulus intensities and the specific muscles targeted, requiring careful interpretation.^31^ Nevertheless, the convergence of evidence strongly implicates reduced IHI as a key supraspinal adaptation, suggesting that cross-education involves an active modulation of the balance between hemispheric excitation and inhibition.

**3.2.3. Motor Cortex Activation Patterns (M1, SMA, PMC)**

Neuroimaging techniques like functional magnetic resonance imaging (fMRI) and positron emission tomography (PET) provide insights into brain regions activated during motor tasks. Studies employing these methods during or after unilateral strength training or contractions have consistently revealed activation not only in the contralateral motor cortex (cM1, relative to the moving limb) but also in the ipsilateral motor cortex (iM1, controlling the resting/untrained limb).^1^ This bilateral activation pattern, particularly the engagement of iM1, is considered a cornerstone of the neural basis for cross-education.^3^ After training, fMRI studies have shown enlarged regions of activation in the iM1 during contractions of the untrained limb.^21^

Beyond the primary motor cortex, other motor-related areas also exhibit bilateral activation and appear crucial for interlimb transfer. Strong evidence points to the involvement of the Supplementary Motor Area (SMA), a region critical for motor planning, sequencing, and interlimb coordination.^25^ One multimodal neuroimaging study demonstrated that functional connectivity between the right and left SMA increased after unilateral ballistic training.^25^ Crucially, the structural integrity of the white matter tracts connecting the bilateral SMAs (assessed using diffusion tensor imaging, DTI) was found to negatively correlate with and predict the magnitude of interlimb transfer across individuals.^25^ This finding strongly suggests that the efficiency of communication between higher-order motor planning areas in the two hemispheres is a significant factor determining the extent of cross-education. Other areas implicated include the premotor cortex (PMC) and the cingulate motor area (CMA), which also show bilateral activity during unilateral movements and may contribute to the network mediating transfer.^25^ Some fMRI evidence has even suggested involvement of temporal lobe regions, potentially related to semantic memory for movement.^21^

While informative, neuroimaging techniques have limitations. The hemodynamic response measured by fMRI (BOLD signal) is an indirect correlate of neural activity and has relatively poor temporal resolution compared to electrophysiological methods.^10^ Distinguishing between excitatory and inhibitory processes or identifying whether activation reflects input, output, or local processing can also be challenging.^10^ Despite these caveats, the consistent finding of bilateral activation, particularly involving iM1 and SMA, provides compelling evidence for supraspinal network involvement in cross-education. The link between SMA connectivity and transfer magnitude specifically implies that cross-education relies not only on primary motor output pathways but also significantly on the integrity and interaction of higher-level motor control centers involved in planning and bilateral coordination.

**3.2.4. Evaluating Theoretical Models: Cross-Activation and Bilateral Access**

Two main theoretical frameworks have been proposed to explain the supraspinal mechanisms of cross-education, primarily focusing on cortical adaptations.^3^

The **"Cross-Activation"** (or "Spillover") model posits that performing a unilateral motor task, especially forceful contractions, leads to concurrent activation of neural circuits in both cerebral hemispheres.^1^ This bilateral activity, driven perhaps by shared command signals or reduced interhemispheric inhibition, is thought to induce simultaneous neural adaptations (e.g., increased excitability, synaptic strengthening) in the pathways controlling both the trained and the untrained limb. Evidence supporting this model includes the fMRI findings of bilateral motor cortex activation ^3^ and the TMS findings of increased iM1 excitability ^3^ and reduced IHI.^4^

The **"Bilateral Access"** (or "Callosal Access") model proposes a different perspective, suggesting that motor learning occurring during unilateral training results in the formation of a motor engram or representation (a learned motor plan or skill component) that is stored in a way that allows it to be accessed and utilized by the motor systems controlling *both* limbs.^1^ This implies that the adaptation is not necessarily occurring simultaneously in both hemispheres during training, but rather that a central representation is formed, which can later be deployed bilaterally, potentially via transcallosal pathways. This model aligns well with the concept of motor learning transfer ^1^ and might be particularly relevant for tasks involving complex sequences or sensorimotor integration.^3^ The findings implicating SMA connectivity in transfer magnitude ^25^ could also fit within this framework, as SMA is involved in accessing and coordinating learned motor plans.

It is widely suggested that these two models are not mutually exclusive and likely represent different facets of a complex process.^1^ The relative contribution of each might depend on the specific demands of the training task. For instance, maximal strength tasks emphasizing high force output might rely more heavily on cross-activation mechanisms, while tasks requiring more precise motor control or learning might engage bilateral access mechanisms more prominently.^3^ The complexity is further highlighted by evidence suggesting that interlimb transfer of certain skills, like prism adaptation, can occur even in individuals with callosal agenesis or lesions, implying that pathways other than the corpus callosum (perhaps subcortical) might also contribute to transfer, at least for some tasks.^52^ This underscores that cross-education likely involves a dynamic interplay between direct interhemispheric influences during training (cross-activation) and the formation and subsequent retrieval of accessible motor representations (bilateral access), potentially utilizing multiple neural pathways. Disentangling the specific contributions and interactions of these processes remains a significant challenge for future research.

### 3.3. Spinal and Subcortical Adaptations Mediating Cross-Education

While cortical adaptations are heavily implicated, evidence also points to the involvement of neural plasticity within the spinal cord and potentially subcortical structures in mediating cross-education of strength.^1^ Although early studies using less sensitive measures sometimes suggested minimal spinal involvement ^2^, more recent investigations employing refined techniques have revealed specific spinal-level adaptations in the untrained limb.

**3.3.1. Spinal Excitability: H-Reflex and V-Wave Findings**

The Hoffmann reflex (H-reflex) is an electrically evoked analog of the monosynaptic stretch reflex, commonly used to assess the excitability of the pathway between Ia afferents and alpha motoneurons, reflecting both presynaptic inhibition acting on Ia terminals and the intrinsic excitability of the motoneuron pool.^3^ Evidence regarding H-reflex changes in the untrained limb following unilateral strength training is notably inconsistent.^15^ Several studies have reported no significant change in the maximal H-reflex amplitude (Hmax) or the Hmax/Mmax ratio (H-reflex normalized to the maximal M-wave) in the untrained homologous muscle.^3^ Conversely, other investigations have observed subtle modulations, such as an increase in H-reflex excitability at the threshold level (suggesting easier recruitment of low-threshold motor units) ^3^, a decrease in Hmax in antagonist muscles (potentially reflecting changes in reciprocal inhibition) ^3^, or even bilateral *reductions* in H-reflex excitability, particularly following training involving fine motor skill components.^26^ Training modality also appears critical, as endurance training tends to increase H-reflex excitability, whereas strength training often does not show the same effect.^48^ Recent work using TMS to condition the H-reflex suggests that changes in polysynaptic pathways influencing motoneuron excitability might occur, particularly after skill-based training.^45^ The discrepancies in H-reflex findings are likely attributable to significant methodological heterogeneity across studies, including differences in the training protocols, muscle groups studied, timing of measurements, and specific H-reflex parameters analyzed.^15^

In contrast to the H-reflex, the volitional wave (V-wave) has yielded more consistent findings. The V-wave is elicited by supramaximal nerve stimulation during a voluntary contraction and is thought to reflect the magnitude of efferent neural drive reaching the muscle from the spinal cord, integrating descending commands and reflex contributions.^1^ Multiple studies have demonstrated an increase in V-wave amplitude (often normalized to Mmax) in the untrained limb following unilateral strength training.^1^ Importantly, increases in V-wave amplitude have been shown to correlate with the magnitude of strength gains in the contralateral limb.^1^ While some researchers propose that V-wave changes are primarily downstream consequences of adaptations at the cortical level (i.e., increased descending drive originating supraspinally) ^3^, the V-wave nonetheless indicates an enhanced ability of the nervous system to effectively activate the motoneuron pool of the untrained muscle during voluntary effort. It is worth noting that acute endurance exercise may decrease V-wave amplitude ^49^, further highlighting the specificity of adaptations to training type.

The divergence between the often-inconsistent H-reflex results and the more consistent V-wave increases is revealing. It suggests that the primary spinal-level adaptation supporting cross-education of *strength* may not be a simple change in the baseline excitability of the Ia-motoneuron reflex pathway (probed by the H-reflex), but rather an enhancement in the *efficacy* of the descending voluntary command in recruiting and driving motoneurons during forceful contractions (better reflected by the V-wave). This enhanced efficacy could result from increased descending input, reduced presynaptic inhibition specifically affecting descending pathways, or altered intrinsic motoneuron properties that make them more responsive to voluntary drive.

**3.3.2. Motoneuron Behaviour: Recruitment and Discharge Properties**

Advances in electromyography, particularly the use of high-density surface EMG (HD-EMG) combined with sophisticated decomposition algorithms, allow for the non-invasive tracking and analysis of individual motor unit (MU) action potentials during voluntary contractions.^7^ Recent studies employing these techniques have provided novel insights into spinal adaptations accompanying cross-education, revealing changes potentially missed by conventional surface EMG.

Specifically, studies have reported significant adaptations in MU behavior within the untrained limb following unilateral resistance training.^4^ These include a decrease in MU recruitment thresholds, meaning that individual motor units are recruited at a lower percentage of maximal force.^4^ This implies that less net excitatory drive is required from the nervous system to bring a given MU to its firing threshold.^4^ Additionally, an increase in the net MU discharge rate (defined as the increase in firing rate from the point of recruitment to the peak rate achieved during a maximal or submaximal contraction) has been observed in the untrained muscles.^4^ A higher net discharge rate suggests a greater capacity for the spinal motoneurons to increase their firing frequency in response to increasing voluntary drive, ultimately contributing to greater force production.^4^

These findings contrast with many earlier studies using conventional surface EMG, which often reported no significant change in the overall amplitude of agonist muscle activity or in average MU firing rates in the untrained limb, leading to the conclusion that spinal adaptations were minimal.^1^ The ability of HD-EMG to resolve the behavior of individual MUs overcomes limitations of global surface EMG signals (e.g., amplitude cancellation) and reveals more nuanced spinal plasticity.^7^ These specific MU adaptations—recruiting units earlier and driving them to higher firing rates—provide a concrete spinal mechanism that directly contributes to the increased force-generating capacity observed in the untrained limb during cross-education.

**3.3.3. Spinal Inhibitory Mechanisms**

Adaptations in spinal inhibitory circuits could also contribute to cross-education, for example, by reducing antagonist muscle co-activation or modulating agonist motoneuron excitability. Evidence for changes in reciprocal inhibition, which coordinates agonist activation with antagonist relaxation, is somewhat limited and indirect. Some studies have reported reductions in antagonist muscle co-activation following unilateral training ^1^, while others found no change.^1^ A decrease in the H-reflex amplitude of the antagonist muscle in the untrained limb was noted in one study, potentially reflecting altered reciprocal inhibition.^3^ Changes in H-reflex excitability of the agonist muscle could also reflect modulation of presynaptic inhibition acting on Ia afferent terminals ^48^, but as discussed, H-reflex findings are inconsistent. Direct evidence for adaptations in specific spinal inhibitory interneurons (e.g., Ia interneurons mediating reciprocal inhibition, Renshaw cells mediating recurrent inhibition) specifically contributing to the cross-education of strength is currently lacking in the reviewed literature.

**3.3.4. Evidence for Subcortical Involvement (Cerebellum, Basal Ganglia)**

While cross-education mechanisms are often conceptualized along the cortico-spinal axis, it is acknowledged that neural adaptations likely occur at multiple levels of the nervous system.^2^ However, the specific roles of subcortical structures, such as the cerebellum and basal ganglia, in mediating the cross-education of *strength* remain largely unexplored in the literature synthesized for this review. These structures are known to be critically involved in motor control, learning, coordination, and scaling of movement parameters. Their involvement in motor skill learning and transfer is more established ^2^, but direct evidence linking them to the transfer of maximal voluntary force production is scarce. It is plausible that these structures contribute, perhaps by refining the motor command transferred from the cortex, integrating sensory feedback, or contributing to the long-term consolidation of adaptations.^31^ However, current research paradigms heavily focused on TMS, fMRI of cortical areas, and spinal reflex measures may not adequately capture the potential contributions of these subcortical loops to strength transfer. One intriguing fMRI finding pointed towards activation changes in the temporal lobe, linked to semantic memory for movement, after unilateral training ^21^, hinting at broader network involvement beyond traditional motor areas. Nonetheless, the potential contribution of the cerebellum and basal ganglia to strength transfer represents a significant knowledge gap in the field.

**Table 1: Summary of Evidence for Proposed Neural Mechanisms Mediating Cross-Education of Strength**

| **Neural Level** | **Proposed Mechanism** | **Key Findings Summary** | **Supporting Techniques** | **Consistency/Strength of Evidence** | **Representative Snippet IDs** |
| --- | --- | --- | --- | --- | --- |
| **Cortical** | Increased Corticospinal Excitability (CSE) in iM1 | Increased MEP amplitude in untrained limb pathway. | TMS | Strong/Consistent | ^3^ |
|  | Reduced SICI in iM1 | Decreased short-interval intracortical inhibition in untrained M1. | TMS (paired-pulse) | Strong/Consistent | ^4^ |
|  | Reduced IHI (Trained → Untrained M1) | Decreased inhibition from trained to untrained hemisphere. | TMS (paired-pulse) | Strong/Consistent | ^11^ |
|  | iM1 Activation | Increased fMRI activation in M1 ipsilateral to trained limb during/after training. | fMRI | Strong/Consistent | ^3^ |
|  | SMA Involvement | Increased bilateral SMA functional connectivity; structural connectivity predicts transfer magnitude. | fMRI, DTI | Moderate/Emerging | ^25^ |
|  | Cross-Activation Model | Bilateral cortical activity during unilateral training drives bilateral adaptations. | Theoretical, supported by fMRI, TMS | Plausible Framework | ^3^ |
|  | Bilateral Access Model | Centrally stored motor engram accessible by both limbs. | Theoretical | Plausible Framework | ^2^ |
| **Spinal** | Increased V-Wave Amplitude | Increased V-wave in untrained limb during MVC, often correlates with strength gain. | V-Wave (EMG + Stimulation) | Moderate/Consistent | ^1^ |
|  | H-Reflex Modulation | Inconsistent findings: often no change in Hmax; some report subtle changes (threshold, antagonist) or reductions. | H-Reflex (EMG + Stimulation) | Limited/Inconsistent | ^3^ |
|  | Decreased MU Recruitment Threshold | Motor units in untrained limb recruited at lower force levels. | HD-EMG Decomposition | Moderate/Emerging | ^7^ |
|  | Increased MU Net Discharge Rate | Motor units in untrained limb show greater increase in firing rate from recruitment to peak. | HD-EMG Decomposition | Moderate/Emerging | ^4^ |
|  | Reciprocal Inhibition Changes | Limited/indirect evidence (e.g., antagonist H-reflex/coactivation changes). | H-Reflex, EMG | Limited/Inconsistent | ^1^ |
| **Subcortical** | Cerebellum/Basal Ganglia Involvement | Minimal direct evidence for role in *strength* transfer; primarily linked to skill. | (Primarily fMRI/PET for skill) | Major Gap | ^3^ |
|  | Temporal Lobe Involvement | Preliminary fMRI evidence suggesting activation changes related to movement memory. | fMRI | Limited/Emerging | ^21^ |

*Note:* iM1 = Motor cortex ipsilateral to the trained limb (contralateral to the untrained limb). CSE = Corticospinal Excitability. SICI = Short-Interval Intracortical Inhibition. IHI = Interhemispheric Inhibition. SMA = Supplementary Motor Area. MEP = Motor Evoked Potential. DTI = Diffusion Tensor Imaging. MU = Motor Unit. HD-EMG = High-Density Electromyography. MVC = Maximal Voluntary Contraction. Hmax = Maximal H-reflex amplitude. Mmax = Maximal M-wave amplitude.

## 4. Discussion: Critical Analysis of Gaps, Limitations, and Inconsistencies

While significant progress has been made in identifying potential neural adaptations contributing to cross-education, a critical analysis reveals substantial methodological challenges, limitations inherent to the investigative techniques, major knowledge gaps, and unresolved inconsistencies within the literature. Addressing these issues is crucial for building a more robust and comprehensive understanding of the phenomenon.

### 4.1. Methodological Challenges in Cross-Education Research

**4.1.1. Study Design and Control Group Issues**

A fundamental challenge, particularly in earlier research, was the lack of adequate control groups.^10^ Many studies failed to account for the potential confounding effects of familiarization with the strength testing procedures, where repeated testing itself can lead to performance improvements unrelated to the training intervention.^10^ Definitive proof of a true contralateral training effect requires comparison to a non-training control group that undergoes the same testing schedule.^10^ Even when control groups are included, some studies report within-group changes in the untrained limb of the training group rather than statistically comparing the change between the training and control groups, thereby forfeiting the benefit of the control.^10^ Furthermore, the majority of studies employ simple pre-post intervention designs. There is a paucity of longitudinal research tracking the time-course of neural and strength adaptations throughout the training period and during detraining or follow-up phases.^4^ This limits understanding of the dynamics of adaptation, including the kinetics of early changes and the persistence of effects. Small sample sizes are also a prevalent issue, often limiting statistical power to detect subtle effects or perform meaningful correlational analyses between neural and behavioral changes, thereby hindering generalizability.^9^

**4.1.2. Sample Heterogeneity and Size Limitations**

Cross-education studies have investigated diverse populations, including healthy young adults, older adults, trained athletes, sedentary individuals, and various clinical cohorts.^1^ While cross-education effects have been demonstrated across different ages and genders ^2^, factors such as age (magnitude may decrease with age ^11^), initial training status, and the presence of neurological or musculoskeletal conditions can influence both the magnitude of strength transfer and the underlying neural adaptations.^3^ This heterogeneity makes it difficult to synthesize findings across studies unless participant characteristics are carefully considered and reported. Compounding this is the aforementioned issue of small sample sizes in many studies ^9^, which not only limits statistical power for detecting main effects but severely restricts the ability to perform subgroup analyses to explore how factors like age or sex might modulate the neural mechanisms of cross-education.

**4.1.3. Variability in Training Protocols and Outcome Measures**

Perhaps one of the most significant challenges is the immense variability in the unilateral resistance training protocols employed across studies.^3^ Interventions differ widely in terms of:

- **Intensity:** Percentage of maximal voluntary contraction (MVC) or repetition maximum (RM).
- **Volume:** Number of repetitions, sets, and sessions per week.
- **Duration:** Total length of the training program (e.g., 3 weeks vs. 12 weeks).
- **Contraction Type:** Isometric, concentric, eccentric, or a combination.
- **Exercise:** Simple single-joint movements (e.g., wrist flexion) vs. more complex multi-joint exercises.
- **Pacing:** Self-paced vs. externally paced (e.g., metronome).

This lack of standardization makes it extremely difficult to compare results directly and to isolate the specific effects of individual training parameters on neural adaptations.^3^ Certain parameters appear particularly influential; for instance, eccentric contractions are frequently reported to elicit larger cross-education effects compared to concentric or isometric training.^3^ The proposed reason often involves the unique neural control strategies and potentially greater afferent feedback associated with eccentric actions.^3^ This consistent observation hints that the nature of sensory input generated during training could be a critical, yet relatively under-investigated, factor modulating the central neural adaptations driving cross-education. However, the precise mechanisms remain unclear, especially as interventions like whole-body vibration, also thought to increase Ia afferent feedback, did not augment strength transfer in one study ^3^, suggesting the pattern or context of afferent input may be crucial. Similarly, externally-paced contractions have been shown to produce large effects and distinct cortical responses compared to self-paced contractions ^3^, possibly due to increased cognitive demand and altered use-dependent plasticity, but direct comparisons in volume-matched studies are needed.^3^

Variability also exists in strength testing methodologies (e.g., isometric vs. dynamic testing, specific device used). The principle of training specificity applies, with strength gains typically being largest when the testing modality matches the training modality.^3^ This highlights the need for careful consideration and reporting of both training and testing procedures. The lack of standardized reporting further complicates meta-analyses and systematic synthesis of the literature.

### 4.2. Limitations and Interpretation Issues of Neurophysiological Techniques

Investigating neural mechanisms relies heavily on indirect measurement techniques, each with inherent limitations that affect data interpretation.

- **TMS:** While invaluable for probing cortical excitability and inhibition non-invasively, TMS measures can be influenced by numerous factors beyond the targeted neural circuits. Results depend on stimulation parameters (intensity, pulse waveform, coil type and orientation), electrode placement, and the participant's level of muscle activation or relaxation and attention during testing.^31^ Interpreting paired-pulse measures like SICI and IHI requires careful control of stimulus intensities.^31^ Furthermore, TMS provides limited spatial resolution and primarily reflects the state of the final corticospinal output pathway or specific intracortical circuits. Distinguishing direct effects from indirect network effects, or separating changes in excitatory versus inhibitory tone based solely on MEP amplitude, can be challenging. Crucially, as noted earlier, changes in resting-state TMS measures (MEP amplitude, SICI, IHI) do not always correlate reliably with the functional outcome of strength gain, raising questions about their direct causal role versus being epiphenomena or reflecting a general state change.^15^
- **fMRI/PET:** These neuroimaging techniques offer good spatial resolution for identifying active brain regions but suffer from poor temporal resolution relative to neural events.^10^ The BOLD signal measured by fMRI is an indirect marker of neural activity, reflecting metabolic demand rather than direct electrical firing. Distinguishing between excitatory and inhibitory activity, or determining whether activation reflects input, output, or local processing within a region, remains challenging.^10^ It can be difficult to ascertain whether observed activation changes are causally related to the strength transfer or are merely associated phenomena.^10^
- **EMG:** Traditional surface EMG provides a global measure of muscle activity but is limited by factors like signal cancellation between positive and negative phases of motor unit action potentials, crosstalk from adjacent muscles, and signal filtering by subcutaneous tissues.^1^ These limitations make it difficult to accurately quantify neural drive or subtle changes in activation patterns, potentially explaining why early studies often failed to detect significant EMG changes in the untrained limb. The advent of HD-EMG allows for decomposition into individual motor unit firing patterns, offering significantly improved resolution for studying spinal output strategies.^4^
- **H-reflex/V-wave:** Spinal reflex measures also present interpretational challenges. The H-reflex amplitude is influenced by the excitability of the motoneuron pool, the efficacy of the Ia afferent synapse, and the level of presynaptic inhibition acting on Ia terminals.^48^ Changes in H-reflex amplitude alone cannot definitively pinpoint the locus of adaptation.^19^ The V-wave is considered a better indicator of functional descending drive during voluntary contraction, but its amplitude also depends on motoneuron recruitment and firing rate modulation, not just supraspinal input.^53^ The marked inconsistencies in H-reflex findings across studies further complicate interpretation.^15^

A general limitation across techniques is the difficulty in directly assessing the contributions of spinal versus supraspinal sites and their interactions.^4^ Most studies measure adaptations at one level (e.g., cortical excitability via TMS) and infer effects at others. Establishing causality from largely correlational data remains a significant hurdle. The inherent limitations of each technique underscore the conclusion that relying on a single method provides an incomplete picture. A more robust understanding necessitates integrating findings from multiple techniques applied within the same study design to cross-validate observations and probe different aspects of the neural adaptation process across multiple levels of the neuroaxis.

### 4.3. Identifying Major Gaps in Current Knowledge

Despite decades of research, several critical gaps remain in our understanding of the neural mechanisms mediating cross-education of strength:

- **Cortico-Spinal Interplay:** The precise interaction and relative weighting of cortical versus spinal adaptations remain poorly defined. How do changes in cortical excitability or network activity translate into specific modifications of spinal motoneuron behavior (recruitment, firing rates) in the untrained limb?.^3^
- **Influence of Training Parameters:** While certain parameters like eccentric contractions appear potent, the specific neural mechanisms underlying their enhanced effect are not fully understood. The role of afferent feedback generated by different contraction types or training paradigms needs systematic investigation.^3^
- **Role of Subcortical Structures:** The contribution of subcortical motor centers, including the basal ganglia and cerebellum, to the transfer of *strength* (as distinct from skill) is largely unknown and represents a major unexplored area.^2^
- **Temporal Dynamics and Persistence:** The detailed time-course of neural adaptations during the initial phase of training and their long-term persistence after training cessation are not well characterized.^4^
- **Specificity (Strength vs. Skill):** While some studies suggest overlapping mechanisms ^1^, the extent to which the neural adaptations mediating strength transfer differ from those mediating skill transfer requires further clarification.^26^
- **Transfer Asymmetry:** Whether the magnitude of cross-education differs depending on whether the dominant or non-dominant limb is trained remains controversial, with conflicting findings in the literature.^11^ The neural basis for any potential asymmetry is unclear.
- **Muscle Sparing Mechanisms:** The specific neural and molecular mechanisms by which cross-education attenuates muscle atrophy and strength loss during limb immobilization warrant deeper investigation.^3^
- **Clinical Translation:** A significant gap exists between laboratory findings and effective clinical implementation. There is a lack of large-scale, high-quality clinical trials demonstrating efficacy and optimal protocols for specific patient populations, particularly those with neurological disorders.^6^ Developing evidence-based guidelines for clinical practice is a critical need.

### 4.4. Addressing Inconsistencies and Contradictory Findings

Several areas within the cross-education literature are marked by inconsistent or contradictory findings, hindering the development of a unified mechanistic understanding. Key examples include:

- **H-Reflex Modulation:** As discussed extensively, findings regarding H-reflex changes in the untrained limb are highly variable, ranging from no change to increases or decreases depending on the study, training type, and parameters measured.^3^ This likely reflects the sensitivity of the H-reflex to diverse factors (presynaptic inhibition, motoneuron excitability, task context) and significant methodological heterogeneity.^15^
- **CSE and Strength Correlation:** The relationship between changes in resting corticospinal excitability (MEP amplitude) or intracortical inhibition (SICI) in iM1 and the magnitude of contralateral strength gain is inconsistent, with several studies finding no significant correlation.^15^ This challenges the assumption that these resting measures directly reflect the functionally relevant adaptation driving strength transfer.
- **Agonist EMG Changes:** While recent HD-EMG studies show clear MU adaptations ^4^, earlier studies using conventional surface EMG often reported no significant increase in agonist EMG amplitude in the untrained limb ^1^, creating conflicting pictures of spinal output changes. This likely reflects methodological limitations of older techniques.
- **Transfer Asymmetry:** Studies investigating whether transfer is greater from the dominant to the non-dominant limb or vice versa have yielded conflicting results, with some showing greater transfer from dominant ^22^, others showing no difference ^22^, and expert consensus remaining undecided.^11^

Resolving these inconsistencies requires careful consideration of the methodological differences between studies (training protocols, measurement techniques, participant populations, statistical power) and acknowledging the inherent complexity and context-dependency of neural plasticity. Future research designed specifically to address these discrepancies, perhaps by directly comparing different training modalities or populations within the same study using standardized, high-resolution techniques, is essential.

## 5. Future Directions for Research

To address the identified gaps and limitations and advance the understanding of neural mechanisms mediating cross-education of strength, future research should focus on methodological rigor, targeted mechanistic questions, and the integration of advanced techniques.

### 5.1. Recommendations for Methodological Advancement

**5.1.1. Standardizing Protocols and Outcome Measures**

A critical step forward requires greater standardization in research methodology to improve comparability across studies and facilitate robust synthesis, such as meta-analyses. Consensus efforts, potentially guided by expert panels like the Delphi study ^11^, should aim to establish core reporting standards for:

- **Training Interventions:** Detailed reporting of exercise type, muscle group, intensity (relative to individual maximum), volume (reps x sets x load), frequency, duration, rest periods, contraction type(s), and pacing (self vs. external).^6^
- **Strength Testing:** Clear description of the testing modality (isometric, isokinetic, dynamic), device used, joint angles, instructions, and familiarization procedures. Using measures specific to the trained action is important.^3^
- **Neurophysiological Measures:** Standardized protocols for TMS (e.g., intensity normalization, coil placement verification, number of trials), fMRI (e.g., task design, analysis pipelines), EMG (e.g., electrode placement, normalization methods), and reflex testing (e.g., background contraction levels, stimulation parameters) are needed. Reporting reliability metrics for measures is encouraged.

Adopting common data elements and standardized reporting checklists could significantly enhance the quality and interpretability of future research.

**5.1.2. Enhancing Study Designs (Longitudinal, Multimodal)**

Future research should move beyond simple pre-post designs and small sample sizes.^9^ Key recommendations include:

- **Robust Control:** Employing randomized controlled trial designs with non-training control groups undergoing identical testing schedules is essential to isolate true training effects.^10^ Active control groups (e.g., performing a different type of training) may also be valuable.
- **Longitudinal Assessment:** Implementing longitudinal designs with multiple measurement time points during the training intervention and during a follow-up detraining period will allow characterization of the time-course of neural adaptations and strength gains, including early-phase kinetics and long-term retention.^4^
- **Multimodal Approaches:** Integrating multiple neurophysiological and neuroimaging techniques within the same study is strongly advocated.^4^ Combining methods that probe different levels of the neuroaxis (e.g., TMS for corticospinal excitability, fMRI for network activation, DTI for structural connectivity, HD-EMG for spinal output) allows for a more comprehensive understanding of the cascade of adaptations and their interrelationships.

**5.1.3. Improving Sample Characterization and Size**

To enhance generalizability and statistical power, future studies should strive for:

- **Larger Sample Sizes:** Adequately powered studies are needed to reliably detect effects and, crucially, to perform meaningful correlational analyses between neural changes and strength outcomes, as well as subgroup analyses.^9^ Power calculations should be reported a priori.
- **Detailed Characterization:** Thorough reporting of participant characteristics (age, sex, handedness, physical activity levels, training history, health status) is necessary to understand sample representativeness and explore potential moderating factors.
- **Diverse Populations:** While foundational research in healthy young adults remains important, expanding investigations to include older adults, different clinical populations (stroke, MS, orthopedic injury, immobilized individuals), and athletes is crucial for understanding the applicability and potential modifications needed for diverse groups.^5^

### 5.2. Key Research Questions to Advance Understanding

Based on the identified gaps, future research should prioritize addressing the following key questions:

1. **Cortico-Spinal Integration:** What is the dynamic interplay between supraspinal (M1, SMA, PMC) and spinal (motoneuron excitability, MU recruitment/firing patterns) adaptations? How do top-down cortical changes drive or modulate specific spinal plasticity in the untrained limb?
2. **Training Parameter Specificity:** How do variations in training intensity, volume, contraction type (especially eccentric vs. others), velocity, pacing, and exercise complexity differentially impact specific neural mechanisms (e.g., IHI, CSE, V-wave, MU behavior)?
3. **Role of Afferent Feedback:** What is the precise contribution of sensory feedback (e.g., from muscle spindles, Golgi tendon organs) generated during unilateral training to the induction and modulation of contralateral neural plasticity? Can manipulating afferent input alter cross-education outcomes?
4. **Subcortical Contributions:** What roles, if any, do the cerebellum, basal ganglia, and thalamic relays play in mediating or modulating the cross-education of *strength*, potentially in coordination, scaling, or consolidation processes?.^31^
5. **Long-Term Adaptations:** What are the neural mechanisms underlying the long-term retention (or decay) of cross-education effects after training cessation?
6. **Strength vs. Skill Transfer:** To what extent do the neural pathways mediating strength transfer overlap with or diverge from those mediating skill transfer? Are there common core mechanisms and task-specific adaptations?.^1^
7. **Transfer Asymmetry:** What neural factors (e.g., hemispheric specialization, baseline IHI differences) underlie the inconsistent findings regarding transfer directionality between dominant and non-dominant limbs?.^11^
8. **Mechanisms of Muscle Sparing:** What are the specific neural signals and downstream molecular pathways within the muscle that mediate the attenuation of disuse atrophy in an immobilized limb via contralateral training?.^3^
9. **Optimizing Clinical Application:** What are the most effective cross-education protocols (dose, type, timing) for specific clinical populations (e.g., stroke severity, time post-injury)? How can cross-education be best integrated with other rehabilitation strategies?

### 5.3. Leveraging Advanced Neuroimaging and Neurophysiological Approaches

Addressing the key research questions will be facilitated by the strategic application and integration of advanced neuroscientific techniques:

- **Network-Level Brain Imaging:** Utilize advanced fMRI analyses like resting-state and task-based functional connectivity, effective connectivity (e.g., Granger causality, dynamic causal modeling), and graph theory approaches to map network interactions (e.g., between M1, SMA, PMC) and how they change with training.^11^ Combine fMRI with DTI to relate functional network changes to underlying white matter structural integrity, particularly of transcallosal pathways.^25^
- **Sophisticated TMS Paradigms:** Move beyond basic MEP and SICI/IHI measures. Employ multi-pulse TMS paradigms to probe different intracortical circuits (e.g., long-interval intracortical inhibition, short-interval intracortical facilitation). Combine TMS with EEG (TMS-EEG) to measure cortical responses and connectivity with high temporal resolution. Use TMS to condition spinal reflexes (e.g., H-reflex) to investigate corticospinal influences on spinal circuits.^45^ Consider using TMS causally (e.g., repetitive TMS or paired associative stimulation) to modulate activity in specific cortical areas (e.g., iM1, SMA) and assess the impact on cross-education, while carefully controlling for potential network effects.^3^
- **High-Resolution Spinal Output Measures:** Continue and expand the use of HD-EMG decomposition techniques to provide detailed quantification of individual motor unit adaptations in the untrained limb, including recruitment thresholds, firing rate modulation, discharge variability, and synchronization patterns.^4^ Correlate these MU parameters with supraspinal measures.
- **Direct Neural Recordings (where feasible):** In specific contexts (e.g., pre-surgical evaluation, animal models informing human studies), microneurography for direct peripheral nerve recordings could provide unparalleled insights into afferent feedback and efferent command signals, though its application in typical human cross-education training studies is limited.

The true potential lies in the thoughtful integration of these techniques within single studies. For instance, identifying cortical network changes with fMRI, probing the excitability of key nodes with TMS, and measuring the functional consequences at the spinal level with HD-EMG allows for tracing the flow of adaptation across the neuroaxis. This multimodal strategy is essential for moving beyond descriptive accounts towards a more mechanistic, causal understanding of how unilateral training reshapes bilateral motor circuits to produce strength gains in the untrained limb.

## 6. Conclusion

### 6.1. Synthesis of Current Understanding

Cross-education of strength, the improvement in force production of an untrained limb following unilateral resistance training of the contralateral limb, is a well-established manifestation of neural plasticity within the human motor system. This scoping review confirms that the phenomenon is mediated by adaptations occurring at multiple levels of the neuroaxis, rather than being attributable to a single locus.

Current evidence strongly implicates supraspinal mechanisms, particularly within the cerebral cortex. Key adaptations include a state of disinhibition in the motor cortex ipsilateral to the trained limb, characterized by increased corticospinal excitability and reduced short-interval intracortical inhibition (SICI). Modulation of interhemispheric inhibition (IHI), typically a reduction from the trained to the untrained hemisphere, also appears crucial, likely facilitating bilateral communication or activation. Neuroimaging highlights the involvement of a network including not only the primary motor cortex (M1) but also higher-order motor areas like the supplementary motor area (SMA), whose interhemispheric connectivity appears related to the magnitude of transfer. Theoretical models of "cross-activation" and "bilateral access" provide plausible, potentially complementary, frameworks for these cortical changes.

Evidence for spinal cord involvement has become more compelling with advanced techniques. While changes in baseline spinal reflex excitability (H-reflex) are inconsistent, measures reflecting the efficacy of descending drive during voluntary contraction (V-wave) consistently show enhancement in the untrained limb. Furthermore, high-density EMG studies reveal specific adaptations in motor unit behavior, including lowered recruitment thresholds and increased discharge rate modulation, providing direct evidence for functional plasticity at the spinal output level. The contribution of subcortical structures remains largely undefined for strength transfer.

Despite significant progress, particularly in identifying candidate mechanisms at cortical and spinal levels, a complete and unified model explaining the precise interplay, relative contributions, and modulation of these adaptations by factors like training parameters remains elusive.

### 6.2. Implications for Neural Plasticity Research and Clinical Practice

Cross-education continues to serve as a valuable and accessible human model for investigating fundamental principles of use-dependent neural plasticity, motor learning, interhemispheric interactions, and the neural basis of strength. Understanding how the nervous system adapts bilaterally in response to unilateral input provides unique insights into the organization and adaptive capacity of the motor system.

The potential for clinical application, especially in rehabilitation settings involving unilateral impairment or immobilization, remains a strong driving force for research in this area.^6^ However, translating laboratory findings into effective and widely adopted clinical practice requires overcoming significant hurdles. These include addressing the methodological limitations outlined in this review, particularly the need for larger, well-controlled studies with standardized protocols and outcome measures, and conducting robust clinical trials specifically designed to evaluate efficacy, optimal dosage, and long-term benefits in target patient populations.^6^ Bridging the gap between research evidence and clinical consensus is paramount.^11^

In conclusion, the study of cross-education of strength has revealed intricate neural adaptations spanning cortical and spinal levels. Future progress hinges on rigorous, methodologically sound research employing integrative, multimodal approaches to disentangle the complex interplay of mechanisms. Continued investigation is essential not only to deepen our fundamental knowledge of neural plasticity but also to unlock the full therapeutic potential of cross-education for individuals facing unilateral motor challenges.

## References

*(Note: A formal reference list would typically format these citations according to a specific style guide like APA or Vancouver. The list above simply provides the identifiers used in the text.)*

1. The cross education of strength and skill following unilateral strength training in the upper and lower limbs - PMC - PubMed Central, accesso eseguito il giorno aprile 11, 2025, [https://pmc.ncbi.nlm.nih.gov/articles/PMC6139459/](https://ouci.dntb.gov.ua/en/)
2. Cross-education of strength and skill: an old idea with applications ..., accesso eseguito il giorno aprile 11, 2025, [https://pmc.ncbi.nlm.nih.gov/articles/PMC4797840/](https://www.researchgate.net/publication/293330458_Increased_cross-education_of_muscle_strength_and_reduced_corticospinal_inhibition_following_eccentric_strength_training)
3. The Cross-Education Phenomenon: Brain and Beyond - PMC - PubMed Central, accesso eseguito il giorno aprile 11, 2025, [https://pmc.ncbi.nlm.nih.gov/articles/PMC5423908/](https://www.josam.org/josam/article/view/54)
4. Cross-education: motor unit adaptations mediate the ... - Frontiers, accesso eseguito il giorno aprile 11, 2025, [https://www.frontiersin.org/journals/physiology/articles/10.3389/fphys.2024.1512309/full](https://pmc.ncbi.nlm.nih.gov/articles/PMC7892509/)
5. Full article: The effect of unilateral training on contralateral limb strength in young, older, and patient populations: a meta-analysis of cross education - Taylor and Francis, accesso eseguito il giorno aprile 11, 2025, [https://www.tandfonline.com/doi/full/10.1080/10833196.2018.1499272](https://www.researchgate.net/publication/246508976_NEURAL_ADAPTATION_TO_RESISTANCE_TRAINING_EVIDENCED_BY_CHANGES_IN_EVOKED_V-WAVE_AND_H_REFLEX_RESPONSES)
6. Exercise prescription and strategies to promote the cross-education of strength: a scoping review - medRxiv, accesso eseguito il giorno aprile 11, 2025, [https://www.medrxiv.org/content/10.1101/2022.09.12.22279860v1.full.pdf](https://journals.physiology.org/doi/10.1152/japplphysiol.00680.2024)
7. Cross-education: motor unit adaptations mediate the strength increase in non-trained muscles following 8 weeks of unilateral resistance training - PubMed Central, accesso eseguito il giorno aprile 11, 2025, [https://pmc.ncbi.nlm.nih.gov/articles/PMC11747592/](https://pmc.ncbi.nlm.nih.gov/articles/PMC10578244/)
8. The cross education of strength and skill following unilateral strength training in the upper and lower limbs - ResearchGate, accesso eseguito il giorno aprile 11, 2025, [https://www.researchgate.net/publication/324604916_The_cross_education_of_strength_and_skill_following_unilateral_strength_training_in_the_upper_and_lower_limbs](https://www.mdpi.com/2075-4663/12/8/224)
9. Effect of Contralateral Strength Training on Muscle Weakness in People With Multiple Sclerosis: Proof-of-Concept Case Series - Oxford Academic, accesso eseguito il giorno aprile 11, 2025, [https://academic.oup.com/ptj/article/96/6/828/2686389](https://www.mdpi.com/2077-0383/12/23/7497)
10. Contralateral effects of unilateral strength training: evidence and ..., accesso eseguito il giorno aprile 11, 2025, [https://journals.physiology.org/doi/full/10.1152/japplphysiol.00531.2006](https://pmc.ncbi.nlm.nih.gov/articles/PMC6139459/)
11. Contralateral Effects of Unilateral Strength and Skill Training: Modified Delphi Consensus to Establish Key Aspects of Cross-Education - PMC - PubMed Central, accesso eseguito il giorno aprile 11, 2025, [https://pmc.ncbi.nlm.nih.gov/articles/PMC7806569/](https://research.bangor.ac.uk/portal/files/22305145/2018_Alenezi_M_PhD.pdf)
12. Cross-Education of Muscular Endurance: A Scoping Review - PMC, accesso eseguito il giorno aprile 11, 2025, [https://pmc.ncbi.nlm.nih.gov/articles/PMC11258191/](https://journals.plos.org/plosone/article/file)
13. A scoping review of the contralateral effects of unilateral peripheral stimulation on neuromuscular function - PubMed Central, accesso eseguito il giorno aprile 11, 2025, [https://pmc.ncbi.nlm.nih.gov/articles/PMC8827438/](https://www.josam.org/josam/article/view/54)
14. A scoping review of the contralateral effects of unilateral peripheral stimulation on neuromuscular function | PLOS One, accesso eseguito il giorno aprile 11, 2025, <https://journals.plos.org/plosone/article?id=10.1371/journal.pone.0263662>
15. Title page Neurophysiological adaptations associated to with the cross-education of muscle strength following chronic unilateral - UCL Discovery, accesso eseguito il giorno aprile 11, 2025, [https://discovery.ucl.ac.uk/id/eprint/10056009/1/Rothwell_Neurophysiological%20adaptations%20in%20the%20untrained%20side%20in%20conjunction%20with%20cross-education%20of%20muscle%20strength_AAM.pdf](https://www.cambridge.org/core/journals/british-journal-of-nutrition/article/systematic-review-and-metaanalysis-of-the-effect-of-protein-and-amino-acid-supplements-in-older-adults-with-acute-or-chronic-conditions/83C8288C73D79685854E78D5415B9029)
16. (PDF) Determining the Effects of Cross-Education on Muscle Strength, Thickness and Cortical Activation Following Limb Immobilization: A Systematic Review and Meta-Analysis - ResearchGate, accesso eseguito il giorno aprile 11, 2025, [https://www.researchgate.net/publication/347610629_Determining_the_Effects_of_Cross-Education_on_Muscle_Strength_Thickness_and_Cortical_Activation_Following_Limb_Immobilization_A_Systematic_Review_and_Meta-Analysis](https://www.frontiersin.org/journals/human-neuroscience/articles/10.3389/fnhum.2016.00204/full)
17. Bilateral transfer of motor performance as a function of motor imagery training: a systematic review and meta-analysis - Frontiers, accesso eseguito il giorno aprile 11, 2025, [https://www.frontiersin.org/journals/psychology/articles/10.3389/fpsyg.2023.1187175/full](https://academic.oup.com/ptj/article/96/6/828/2686389)
18. (PDF) Cross‐education of lower limb muscle strength following resistance exercise training in males and females: A systematic review and meta‐analysis - ResearchGate, accesso eseguito il giorno aprile 11, 2025, [https://www.researchgate.net/publication/383788000_Cross-education_of_lower_limb_muscle_strength_following_resistance_exercise_training_in_males_and_females_A_systematic_review_and_meta-analysis](https://journals.physiology.org/doi/full/10.1152/japplphysiol.00802.2011)
19. Neural adaptations underlying cross-education after unilateral strength training, accesso eseguito il giorno aprile 11, 2025, <https://www.researchgate.net/publication/26814505_Neural_adaptations_underlying_cross-education_after_unilateral_strength_training>
20. (PDF) The Cross-Education Phenomenon: Brain and Beyond - ResearchGate, accesso eseguito il giorno aprile 11, 2025, [https://www.researchgate.net/publication/317139457_The_Cross-Education_Phenomenon_Brain_and_Beyond](https://pmc.ncbi.nlm.nih.gov/articles/PMC11258191/)
21. Neuro-Physiological Adaptations Associated with Cross-Education of Strength, accesso eseguito il giorno aprile 11, 2025, [https://www.researchgate.net/publication/5911973_Neuro-Physiological_Adaptations_Associated_with_Cross-Education_of_Strength](https://www.fisiologiadelejercicio.com/wp-content/uploads/2025/01/Comparison-of-Muscle-Growth-and-Dynamic-Strength-Adaptations.pdf)
22. Cross-Education of Strength Depends on Limb Dominance: Implications for Theory and Application | Request PDF - ResearchGate, accesso eseguito il giorno aprile 11, 2025, [https://www.researchgate.net/publication/40443009_Cross-Education_of_Strength_Depends_on_Limb_Dominance_Implications_for_Theory_and_Application](https://www.eneuro.org/content/8/4/ENEURO.0190-20.2021)
23. Exercise prescription and strategies to promote the cross-education of strength: a scoping review - Canadian Science Publishing, accesso eseguito il giorno aprile 11, 2025, [https://cdnsciencepub.com/doi/abs/10.1139/apnm-2023-0041](https://pmc.ncbi.nlm.nih.gov/articles/PMC11747592/)
24. Effects of Cross-Education on Neural Adaptations Following Non-Paretic Limb Training in Stroke: A Scoping Review with Implications for Neurorehabilitation - ResearchGate, accesso eseguito il giorno aprile 11, 2025, [https://www.researchgate.net/publication/362571282_Effects_of_Cross-Education_on_Neural_Adaptations_Following_Non-Paretic_Limb_Training_in_Stroke_A_Scoping_Review_with_Implications_for_Neurorehabilitation](https://www.mdpi.com/2076-3425/13/4/679)
25. Structural and Functional Cortical Connectivity Mediating Cross Education of Motor Function, accesso eseguito il giorno aprile 11, 2025, [https://www.jneurosci.org/content/37/10/2555](https://www.researchgate.net/publication/40443009_Cross-Education_of_Strength_Depends_on_Limb_Dominance_Implications_for_Theory_and_Application)
26. 1894 revisited: Cross-education of skilled muscular control in women and the importance of representation - PubMed Central, accesso eseguito il giorno aprile 11, 2025, [https://pmc.ncbi.nlm.nih.gov/articles/PMC8929574/](https://www.tandfonline.com/doi/full/10.1080/10833196.2018.1499272)
27. Evaluation of Contralateral Limb Cross Education and High-Frequency Repetitive Transcranial Magnetic Stimulation on Functional Indices of the Affected Upper Limb in Subacute Phase of Stroke - PubMed Central, accesso eseguito il giorno aprile 11, 2025, [https://pmc.ncbi.nlm.nih.gov/articles/PMC10751172/](https://www.medrxiv.org/content/10.1101/2022.09.12.22279860v1.full.pdf)
28. Mirror training to augment cross-education during resistance training: a hypothesis - Frontiers, accesso eseguito il giorno aprile 11, 2025, [https://www.frontiersin.org/journals/human-neuroscience/articles/10.3389/fnhum.2013.00396/full](https://discovery.ucl.ac.uk/id/eprint/10056009/1/Rothwell_Neurophysiological%20adaptations%20in%20the%20untrained%20side%20in%20conjunction%20with%20cross-education%20of%20muscle%20strength_AAM.pdf)
29. editor-in-chief - eduCAPES, accesso eseguito il giorno aprile 11, 2025, <https://educapes.capes.gov.br/bitstream/capes/747532/1/Collection%20of%20international%20topics%20in%20health%20sciences.pdf>
30. Determining the Effects of Cross-Education on Muscle Strength, Thickness and Cortical Activation Following Limb Immobilization: - The Journal of Science and Medicine, accesso eseguito il giorno aprile 11, 2025, [https://www.josam.org/josam/article/view/54?download=pdf](https://www.researchgate.net/publication/324604916_The_cross_education_of_strength_and_skill_following_unilateral_strength_training_in_the_upper_and_lower_limbs?download=pdf)
31. Neural pathways mediating cross education of motor ... - Frontiers, accesso eseguito il giorno aprile 11, 2025, [https://www.frontiersin.org/journals/human-neuroscience/articles/10.3389/fnhum.2013.00397/full](https://www.frontiersin.org/journals/physiology/articles/10.3389/fphys.2024.1512309/full)
32. Determining the Effects of Cross-Education on Muscle Strength, Thickness and Cortical Activation Following Limb Immobilization: A Systematic Review and Meta-Analysis | The Journal of Science and Medicine, accesso eseguito il giorno aprile 11, 2025, [https://www.josam.org/josam/article/view/54](https://journals.physiology.org/doi/full/10.1152/japplphysiol.00531.2006)
33. Cross-education Mechanisms and Clinical Rehabilitation Research: A Literature Review | Quality in Sport - Akademicka Platforma Czasopism, accesso eseguito il giorno aprile 11, 2025, [https://apcz.umk.pl/QS/article/view/56330](https://pmc.ncbi.nlm.nih.gov/articles/PMC4797840/)
34. Potential Importance of Maximal Upper Body Strength-Generating Qualities and Upper Body Strength Training for Performance of High-Intensity Running and Jumping Actions: A Scoping Review - PMC - PubMed Central, accesso eseguito il giorno aprile 11, 2025, [https://pmc.ncbi.nlm.nih.gov/articles/PMC11679821/](https://www.frontiersin.org/journals/psychology/articles/10.3389/fpsyg.2023.1187175/full)
35. Is tDCS an Adjunct Ergogenic Resource for Improving Muscular Strength and Endurance Performance? A Systematic Review - PubMed Central, accesso eseguito il giorno aprile 11, 2025, <https://pmc.ncbi.nlm.nih.gov/articles/PMC6532530/>
36. (PDF) Cross-Education of Muscular Endurance: A Scoping Review - ResearchGate, accesso eseguito il giorno aprile 11, 2025, <https://www.researchgate.net/publication/380665977_Cross-Education_of_Muscular_Endurance_A_Scoping_Review>
37. Biomechanical and Physiological Variables in Dynamic and Functional Balance Control during Single-Leg Loading in Individuals with Chronic Ankle Instability: A Scoping Review - MDPI, accesso eseguito il giorno aprile 11, 2025, [https://www.mdpi.com/2075-4663/12/8/224](https://medicina.us.es/sites/medicina/files/doc/Trabajo%20Premiado_3.pdf)
38. Full article: The role of menstrual cycle phase-based resistance training for women post anterior cruciate ligament reconstruction: a scoping review, accesso eseguito il giorno aprile 11, 2025, [https://www.tandfonline.com/doi/full/10.1080/10833196.2023.2266320](https://pmc.ncbi.nlm.nih.gov/articles/PMC10751172/)
39. OUCI, accesso eseguito il giorno aprile 11, 2025, [https://ouci.dntb.gov.ua/en/?backlinks_to=10.1016/j.apmr.2020.12.012](https://www.researchgate.net/publication/317139457_The_Cross-Education_Phenomenon_Brain_and_Beyond?backlinks_to=10.1016/j.apmr.2020.12.012)
40. Increased cross-education of muscle strength and reduced corticospinal inhibition following eccentric strength training | Request PDF - ResearchGate, accesso eseguito il giorno aprile 11, 2025, [https://www.researchgate.net/publication/293330458_Increased_cross-education_of_muscle_strength_and_reduced_corticospinal_inhibition_following_eccentric_strength_training](https://nursing.ceconnection.com/files/NurseAssistedRehabilitationProtocolsFollowingAnteriorCruciateLigamentReconstruction-1718650860911.pdf)
41. Vertical Strength Transfer Phenomenon Between Upper Body and Lower Body Exercise: Systematic Scoping Review - PubMed Central, accesso eseguito il giorno aprile 11, 2025, [https://pmc.ncbi.nlm.nih.gov/articles/PMC11329601/](https://www.jneurosci.org/content/37/10/2555)
42. Corticospinal adaptations following resistance training and its relationship with strength - Facultad de Medicina, accesso eseguito il giorno aprile 11, 2025, [https://medicina.us.es/sites/medicina/files/doc/Trabajo%20Premiado_3.pdf](https://www.researchgate.net/publication/383788000_Cross-education_of_lower_limb_muscle_strength_following_resistance_exercise_training_in_males_and_females_A_systematic_review_and_meta-analysis)
43. synaptoneurosomes identifies neuroplasticity: Topics by Science.gov, accesso eseguito il giorno aprile 11, 2025, <https://www.science.gov/topicpages/s/synaptoneurosomes+identifies+neuroplasticity.html>
44. The cross education of strength and skill following unilateral strength training in the upper and lower limbs - American Journal of Physiology, accesso eseguito il giorno aprile 11, 2025, [https://journals.physiology.org/doi/full/10.1152/jn.00116.2018](https://www.tandfonline.com/doi/full/10.1080/10833196.2023.2266320)
45. Full article: Acute Effects of Strength and Skill Training on the Cortical and Spinal Circuits of Contralateral Limb - Taylor & Francis Online, accesso eseguito il giorno aprile 11, 2025, [https://www.tandfonline.com/doi/full/10.1080/00222895.2023.2265316](https://cdnsciencepub.com/doi/abs/10.1139/apnm-2023-0041)
46. Relative Neuroadaptive Effect of Resistance Training along the Descending Neuroaxis in Older Adults - MDPI, accesso eseguito il giorno aprile 11, 2025, [https://www.mdpi.com/2076-3425/13/4/679](https://www.researchgate.net/publication/347610629_Determining_the_Effects_of_Cross-Education_on_Muscle_Strength_Thickness_and_Cortical_Activation_Following_Limb_Immobilization_A_Systematic_Review_and_Meta-Analysis)
47. Objectivizing Measures of Post-Stroke Hand Rehabilitation through Multi-Disciplinary Scales, accesso eseguito il giorno aprile 11, 2025, [https://www.mdpi.com/2077-0383/12/23/7497](https://pmc.ncbi.nlm.nih.gov/articles/PMC7806569/)
48. Changes in H reflex and V wave following short-term endurance and strength training, accesso eseguito il giorno aprile 11, 2025, [https://journals.physiology.org/doi/full/10.1152/japplphysiol.00802.2011](https://www.tandfonline.com/doi/full/10.1080/00222895.2023.2265316)
49. Changes in H-reflex, V-wave, and contractile properties of the plantar flexors following concurrent exercise sessions—the acute interference effect - American Journal of Physiology, accesso eseguito il giorno aprile 11, 2025, [https://journals.physiology.org/doi/10.1152/japplphysiol.00680.2024](https://pmc.ncbi.nlm.nih.gov/articles/PMC5423908/)
50. 1894 revisited: Cross-education of skilled muscular control in women and the importance of representation - PLOS, accesso eseguito il giorno aprile 11, 2025, [https://journals.plos.org/plosone/article/file?type=printable&id=10.1371/journal.pone.0264686](https://www.frontiersin.org/journals/human-neuroscience/articles/10.3389/fnhum.2013.00397/full?type=printable&id=10.1371/journal.pone.0264686)
51. Neural Adaptations Associated with Interlimb Transfer in a Ballistic Wrist Flexion Task, accesso eseguito il giorno aprile 11, 2025, [https://www.frontiersin.org/journals/human-neuroscience/articles/10.3389/fnhum.2016.00204/full](https://www.medrxiv.org/content/10.1101/2022.12.18.22282724v1.full-text)
52. Interlimb Transfer of Reach Adaptation Does Not Require an Intact Corpus Callosum: Evidence from Patients with Callosal Lesions and Agenesis | eNeuro, accesso eseguito il giorno aprile 11, 2025, [https://www.eneuro.org/content/8/4/ENEURO.0190-20.2021](https://apcz.umk.pl/QS/article/view/56330)
53. NEURAL ADAPTATION TO RESISTANCE TRAINING EVIDENCED BY CHANGES IN EVOKED V-WAVE AND H REFLEX RESPONSES | Request PDF - ResearchGate, accesso eseguito il giorno aprile 11, 2025, [https://www.researchgate.net/publication/246508976_NEURAL_ADAPTATION_TO_RESISTANCE_TRAINING_EVIDENCED_BY_CHANGES_IN_EVOKED_V-WAVE_AND_H_REFLEX_RESPONSES](https://www.frontiersin.org/journals/human-neuroscience/articles/10.3389/fnhum.2013.00396/full)
54. Nurse-Assisted Rehabilitation Protocols Following Anterior Cruciate Ligament Reconstruction - CEConnection, accesso eseguito il giorno aprile 11, 2025, [https://nursing.ceconnection.com/files/NurseAssistedRehabilitationProtocolsFollowingAnteriorCruciateLigamentReconstruction-1718650860911.pdf](https://www.researchgate.net/publication/5911973_Neuro-Physiological_Adaptations_Associated_with_Cross-Education_of_Strength)
55. Motor Memory Consolidation Deficits in Parkinson's Disease: A Systematic Review with Meta-Analysis - PubMed Central, accesso eseguito il giorno aprile 11, 2025, [https://pmc.ncbi.nlm.nih.gov/articles/PMC10578244/](https://pmc.ncbi.nlm.nih.gov/articles/PMC11679821/)
56. Motor Skill Retention Impairments in Parkinson's Disease: A Systematic Review with Meta-analysis | medRxiv, accesso eseguito il giorno aprile 11, 2025, [https://www.medrxiv.org/content/10.1101/2022.12.18.22282724v1.full-text](https://pmc.ncbi.nlm.nih.gov/articles/PMC8929574/)
57. UCF Ph.D. in Kinesiology - State University System of Florida, accesso eseguito il giorno aprile 11, 2025, <https://www.flbog.edu/wp-content/uploads/2021/10/ASA_04bii_UCF_PhD_Kinesiology_Proposal_CE.pdf>
58. Bangor University DOCTOR OF PHILOSOPHY Motor imagery as a potential tool for improvement of musculoskeletal function in physiotherapy practice Alenezi, Majid - Research Portal, accesso eseguito il giorno aprile 11, 2025, [https://research.bangor.ac.uk/portal/files/22305145/2018_Alenezi_M_PhD.pdf](https://www.researchgate.net/publication/362571282_Effects_of_Cross-Education_on_Neural_Adaptations_Following_Non-Paretic_Limb_Training_in_Stroke_A_Scoping_Review_with_Implications_for_Neurorehabilitation)
59. paralyzed leg muscles: Topics by Science.gov, accesso eseguito il giorno aprile 11, 2025, <https://www.science.gov/topicpages/p/paralyzed+leg+muscles>
60. Systematic review and meta-analysis of the effect of protein and amino acid supplements in older adults with acute or chronic conditions | British Journal of Nutrition - Cambridge University Press & Assessment, accesso eseguito il giorno aprile 11, 2025, [https://www.cambridge.org/core/journals/british-journal-of-nutrition/article/systematic-review-and-metaanalysis-of-the-effect-of-protein-and-amino-acid-supplements-in-older-adults-with-acute-or-chronic-conditions/83C8288C73D79685854E78D5415B9029](https://pmc.ncbi.nlm.nih.gov/articles/PMC11329601/)
61. Comparison of Muscle Growth and Dynamic Strength Adaptations Induced by Unilateral and Bilateral Resistance Training: A Systemat - Fisiología del Ejercicio, accesso eseguito il giorno aprile 11, 2025, [https://www.fisiologiadelejercicio.com/wp-content/uploads/2025/01/Comparison-of-Muscle-Growth-and-Dynamic-Strength-Adaptations.pdf](https://pmc.ncbi.nlm.nih.gov/articles/PMC8827438/)
62. The knowns and unknowns of neural adaptations to resistance training - PMC, accesso eseguito il giorno aprile 11, 2025, [https://pmc.ncbi.nlm.nih.gov/articles/PMC7892509/](https://journals.physiology.org/doi/full/10.1152/jn.00116.2018)
